# Developmental neurotoxicity of 3,3’,4,4’-tetrachloroazobenzene with thyroxine deficit: Sensitivity of glia and dentate granule neurons in the absence of behavioral changes

**DOI:** 10.3390/toxics2030496

**Published:** 2014-09-24

**Authors:** G. Jean Harry, Michelle J. Hooth, Molly Vallant, Mamta Behl, Gregory S. Travlos, James L. Howard, Catherine J. Price, Sandra McBride, Ron Mervis, Peter R. Mouton

**Affiliations:** 1 Neurotoxicology Group, National Toxicology Program Laboratory, NIEHS, RTP, NC; 2 Division of the National Toxicology Program, NIEHS, RTP, NC; 3 Howard Associates, RTP, NC; 4 RTI International, RTP, NC; 5 Social and Scientific Systems, Inc., Durham, NC; 6 NeuroStructural Research Labs, Inc, Tampa, FL; 7 Department of Pathology & Cell Biology, University of South Florida School of Medicine, Tampa, FL; 8 Stereology Resource Center, Tampa, FL

**Keywords:** astrocytes, thyroid hormone, developmental neurotoxicology, microglia, Golgi Purkinje cells, dentate granule cells, dioxin, hippocampus, glial fibrillary acidic protein

## Abstract

Thyroid hormones (TH) regulate biological processes implicated in neurodevelopmental disorders and can be altered with environmental exposures. Developmental exposure to the dioxin-like compound, 3,3’,4,4’-tetrachloroazobenzene (TCAB), induced a dose response deficit in serum T4 levels with no change in 3,5,3’- triiodothyronine or thyroid stimulating hormone. Female Sprague-Dawley rats were orally gavaged (corn oil, 0.1, 1.0, or 10 mg TCAB/kg/day) two weeks prior to cohabitation until post-partum day 3 and male offspring from post-natal day (PND)4-21. At PND21, the high dose showed a deficit in body weight gain. Conventional neuropathology detected no neuronal death, myelin disruption, or gliosis. Astrocytes displayed thinner and less complex processes at 1.0 and 10 mg/kg/day. At 10 mg/kg/day, microglia showed less complex processes, unbiased stereology detected fewer hippocampal CA1 pyramidal neurons and dentate granule neurons (GC) and Golgi staining of the cerebellum showed diminished Purkinje cell dendritic arbor. At PND150, normal maturation of GC number and Purkinje cell branching area was not observed in the 1.0 mg/kg/day dose group with a diminished number and branching suggestive of effects initiated during developmental exposure. No effects were observed on post-weaning behavioral assessments in control, 0.1 and 1.0mg/kg/day dose groups. The demonstrated sensitivity of hippocampal neurons and glial cells to TCAB and T4 deficit raises support for considering additional anatomical features of brain development in future DNT evaluations.

## 1. Introduction

The complexity of the nervous system and the ability to examine endpoints reflecting integrated functions has lead to a reliance on neurobehavioral evaluations in neurotoxicity assessments and thus, are prominent within standard developmental neurotoxicity (DNT) studies. However, questions have been raised over the years regarding the sensitivity of such methods, as conducted, to detect low-dose developmental neurotoxicity, subtle effects due to hormonal disruption (e.g. thyroid hormone), or long-lasting effects in the adult following compensatory responses. Based on these concerns, efforts have been proposed to refine testing protocols, enhance the amount of quality data generated, identify endpoints selectively sensitive to the known effects of compounds, and integrate advances in basic neurobiology into the neurotoxicity testing strategy.

Thyroid hormone is a critical regulator of biological processes essential for brain development and implicated in various neurodevelopmental disorders [1-5]. Deficiency, even of short duration, has been linked with irreversible brain damage, depending on timing of onset and duration [6]. The majority of experimental animal studies employed to examine effects of TH disruption utilize models of known goitrogens (e.g., propylthiouracil (PTU), methimazole, or iodine deficiency) as positive controls for inhibiting production of TH. These classic goitrogens inhibit thyroid peroxidase (TPO), the enzyme responsible for TH formation in the thyroid [7]. With TPO inhibition, synthesis of new TH is reduced as a result of a deficit in iodine transference to thyroglobin, quickly decreasing levels of serum TH. Thyroiditis-induced hypothyroidism usually occurs when the thyroid gland cannot produce thyroxine (T4), resulting in decreased serum T4 levels and increased thyroid stimulating hormone (TSH). Alternatively, it can be induced by disruption of the feedback cycle of thyroid-releasing hormone, low TSH secretion, and altered thyroid stimulation or as a result of increased hormonal removal via elevated liver glucuronidation [8].

T4 requires conversion to T3 for binding of the TH receptor and downstream signaling. While plasma T3 can be transported into the brain [9], T4 uptake into the brain is critical for normal T3- mediated processes [10]. Thus, any change in serum T4 levels would be expected to significantly influence such processes. Approximately 80% of active T3 is generated *in situ* from T4 deiodination by type 2-iodothyronine deiodinase [11]. During hypothyroidism, the brain maintains T3 levels by increasing type-II 5-deiodinase (Dio2) expression and activity in astrocytes [12]. While increased dio2 mRNA levels and activity are observed in various brain regions, the hippocampus and cerebral cortex showed the greatest level of sensitivity [13]. Importantly, the fetus is closely dependent upon the maternal compartment for T4 and deiodination, and T4 serves as the precursor for the required T3 in the fetal rat brain [14-15]. Even mild maternal hypothyroxinemia in humans, defined as low serum T4 levels with normal T3 and TSH levels, has been associated with neurodevelopmental disorders [16]. Several classes of environmental chemicals act as TH disruptors through different mechanisms often leading to hypothyroxinemia, [17-20]. The range of thyroid disrupting chemicals is relatively broad, encompassing industrial chemicals like polychlorinated biphenyls (PCBs), dioxins, and flame-retardants, as well as, pesticides or ingredients in personal care products [21-24]. In many cases of chemical-associated hypothyroxinemia, disruption is transient and levels recover with cessation of exposure; however, at a sufficient level of deficit or critical ages, long-term effects on the nervous system have been reported [25]. Thus, interference with thyroid function or TH action is likely an important mechanism by which some environmental contaminants may produce neurotoxic effects.

Given the large number and varied classes of chemicals that can significantly affect the TH system, this mode of action has been considered for potential to underlie multiple aspects of neurotoxicity. Severe TH dysfunction in the rat induced by known goitrogens can produce anatomical effects on brain development and functional changes in startle reactivity, auditory acuity, and spatial learning [26]. However, while such effects have established an expected characteristic profile for developmental TH disruption, a broad and relatively severe effect on the TH system including T4, T3, and TSH, appears to be required. Quite often the characteristic profile is not identified following mild to moderate levels of TH deficiency or hypothyroxinemia. While robust endpoints for chemical induced TH deficits of approximately 50% are associated with hippocampal [27] or auditory [28] physiological activation, even at these levels of deficit there is a general paucity of neurobehavioral or overt neuroanatomical effects [26]. This lack of effect on standard neurotoxicity screening endpoints continues to hinder the ability of DNT studies to adequately evaluate the impact of developmental T4 deficit as occurs with chemical exposure. Given that T4 is the primary TH utilized for T3 production in the brain, the overall lack of sensitivity for standard DNT tests to detect neurotoxicity of such TH disruptors has promoted efforts to identify new and more sensitive endpoints.

Dioxin and dioxin-like compounds fall within the classification of chemicals that induce a disruption to the developing TH system, show some signs of altering cellular processes important in the nervous system yet, quite often fail to manifest as alterations in behavior [29]. To explore possible alternative or expanded endpoints for inclusion into a developmental neurotoxicity assessment to evaluate the impact of T4 deficit occurring from chemical exposure, we utilized the compound 3,3’,4,4’-tetrachloroazobenzene (TCAB). TCAB is a by-product formed in the manufacture of herbicidal derivatives and with the degradation of chloroanilide herbicides. It structurally resembles 2,3,7,8-tetrachlorodibenzo-p-dioxin yet, binds to the aryl hydrocarbon receptor with an approximate 1/5^th^ affinity [30-31]. Numerous studies have reported that TCAB exhibits both dioxin-like effects and chemical-specific properties including a significant reduction in serum T4 concentrations with little or no concomitant increase in circulating TSH or evidence of thyroid gland histopathology [32-35]. So, while TCAB could have direct effects upon the developing nervous system, the significant level of T4 deficit observed focused our evaluation on endpoints to detect changes that would be hypothesized to occur based on available data from developmental exposure to known goitrogens as well as standard developmental neurotoxicity assessments. Using a developmental exposure regimen for TCAB encompassing gestational and lactational periods, we examined numerous neurobehavioral endpoints and anatomical features of brain development [36-42] that have been previously associated with developmental deficits in TH.

## 2. Experimental Section

Chemical verification, dose formulation and stability, animal assignment, dosing, pre-weaning endpoints, locomotor activity, tissue collection conducted by Research Triangle Institute (RTI), tissue processing by Neurosciences Associates (Knoxville, TN), and qualitative pathology evaluation provided by Midwest ToxPath Sciences, Inc, (Chesterfield, MO) was carried out in compliance with the U.S. Food and Drug Administration, Good Laboratory Practice regulations, 21 DRF 58.

### 2.1. Animals

**Figure 1:**
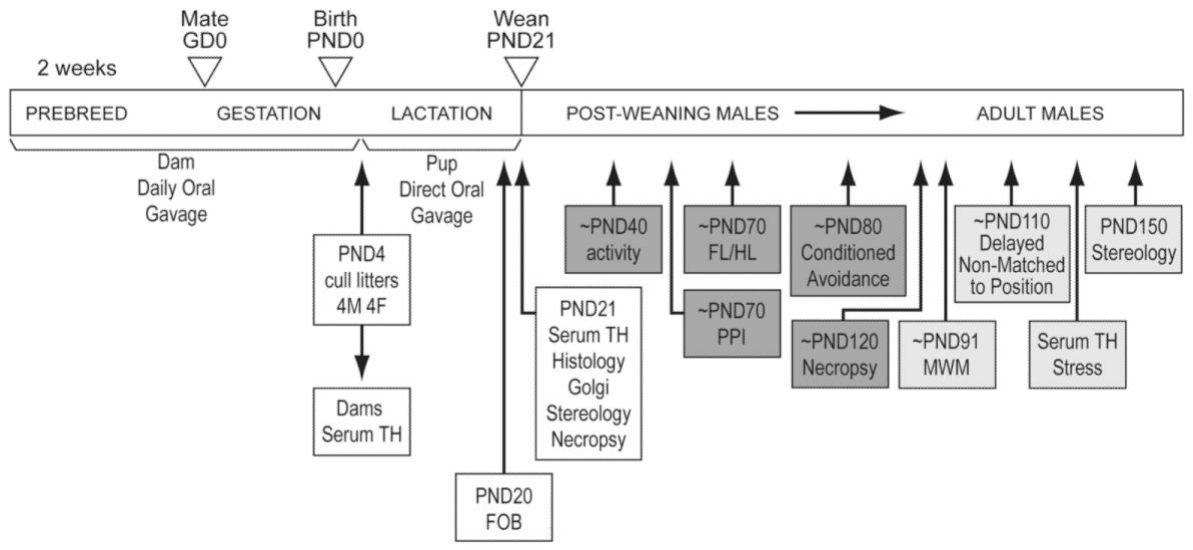
Schematic of dosing and allocation of animals to terminal and behavioral endpoints Female rats were dosed by oral gavage for 2 weeks prior to breeding and throughout gestation (GD0) until post-partum day 3. The pups were dosed by oral gavage from postnatal day (PND)4 until PND21. Pups were randomly assigned to groups identified for testing between PND40-80 and then used for PND120 necropsy or pups were used for behavioral assessments PND90 through to PND150 for unbiased stereology.

Female Sprague-Dawley rats (Charles River Laboratories, Raleigh, NC; were randomly assigned to vehicle (1% acetone/99% corn oil) or 0.1, 1.0, 10 mg/kg/day TCAB (Accu Standard, New Haven, CT)/kg body weight (n=20-25/group) and received oral gavage (5ml/kg body wt/day) for 2 weeks prior to mating through postnatal day (PND)3. The dose regimen was selected based upon previous studies with TCAB [34-35] and included a pre-implantation and gestational period followed by direct dosing to the offspring to maintain accurate dose level delivery. Chemical verification, dose formulation and stability were confirmed by infrared, nuclear magnetic resonance and low-resolution mass spectrometry by methods previously described [43]. To generate a minimum of 11 litters per dose group with matched birthdates, 20 dams per control, 0.1 and 1.0 mg/kg/day TCAB and 30 for 10 mg/kg/day TCAB were initially placed on study. From these litters, 11 litters per dose group were randomly selected and were culled to 4 males/4 females on PND4 and males assigned to specific endpoints (Fig. 1) while females were utilized in a separate study. These pups then received a direct oral dosing via a micropipette with plastic tip progressing with age to a 20-22g stainless steel feeding tube until PND21 with the same vehicle or dose level received by the dam. Rats were singularly housed in a semi-barrier facility (21±2°C; 50±10% relative humidity; 12-hr light/dark cycle (6:00-18:00); 30±3 foot candles normal room lighting). Water met National Toxicology Program drinking water requirements [44] and food was available *ad libitum.* All procedures were conducted in accordance with RTI International’s IACUC approved animal protocol.

### 2.2. Physical Parameters and Functional Observational Battery (FOB)

Dam body weight was recorded weekly prior to mating, daily between GD0 and post-partum day 3, and on post-partum days 4, 7, 14, and 21. Pup body weights were recorded daily from PND4-21 and weekly thereafter until termination. Adverse signs of toxicity were recorded at time of dosing and between 1-2 hrs post-dosing. On PNDs 1-10, maternal behavior (nesting, pup retrieval, nursing and pup milk band) was recorded daily. All pups within a litter were examined for physical development parameters daily until observed (pinna detachment (PND1-4), righting reflex in hand (PND4-9), incisor eruption (PND8-16), eye opening (PND11-16), nipple retention, hypospadias, testicular descent (PND16-20) daily. Beginning on PND35, preputial separation was examined until acquisition. On PND20, one previously assigned male per litter (n=10/group) was assessed using a modified FOB. Rats were assessed for handling reactivity, changes in general appearance including, lacrimation, salivation, ptosis, pupil size, and piloerection. When placed on a flat surface, activity level, arousal, posture, gait, and occurrence of involuntary motor movements (e.g., tremors, convulsions) were scored for 2 min. Response to an auditory stimuli (click response), tail-pinch, pupil constriction to penlight stimuli was recorded. Aerial righting and landing foot splay were recorded following a drop of 30 cm. Forelimb grabbing was assessed as the ability to grasp an inverted screen for a total of 5 sec. Hindlimb grip strength was quantified using a digital force strain gauge (Chatillon, AMETEK, Inc., Largo, FL) fitted with a T-bar and attached to a trough platform. One experimenter blinded with respect to individual animal dose group performed assessments.

### 2.3. TH Analysis

At post-partum day 4, a subset of dams for which litters were not continued for neurotoxicity evaluation, and pups at PND21 (3-hrs post-dosing) were deeply anesthetized (70% CO_2_), blood collected via cardiac puncture, and serum stored at −80°C. One week following adult behavioral testing, in an effort to unmask underlying changes in TH levels, adult littermates were randomly assigned to either a non-stressed (home-cage) or stressed (15-min restraint within a semi-cylindrical restrainer) group. At cessation of restraint, retro-orbital blood (isoflurane anesthesia) was collected for T4 analysis. Serum T3, T4, and TSH analyses were performed in dams and serum T4 in adult males using radioimmunoassay (Apex Automatic Gamma Counter; Huntsville, AL) with reagents for T3 and T4 (Siemens Healthcare Diagnostics, Los Angeles, CA) and rat-specific TSH analyses (American Laboratory Products, Windham, NH). Due to the limited serum sample volume at PND21, serum T3, T4 and TSH analyses were performed using a fluorescent-coded, bead-based, multiplex immunoassay method (LiquiChip 200; QIAGEN, Valencia, CA) and rat-specific reagents (Millipore Corporation; Billerica, MA). For comparison, T4 levels at PND21 were also analyzed by the radioimmunoassay procedure used in adults.

### 2.4. Behavioral Assessments

Previous studies have demonstrated that severe developmental disruptions of TH function by known goitrogens (PTU, iodine deficiency) can lead to anatomical changes in the brain and alterations in numerous neurobehavioral functions including activity, startle reactivity, and learning and memory [45-46]. These findings however have been predominantly linked to hypothyroidism with decreased serum levels of T4 and T3 with a concurrent increase in TSH. In the case of hypothyroxinemia, the manifestation of behavioral changes due to developmental disruption appears to be less prominent. This is not limited to hypothyroxinemia but rather can also be observed with known goitrogens depending on the level of severity of the TH disruption. As an example, in a model of developmental iodine deficiency, a deficit in T4 of up to 60% failed to produce effects on standard DNT neurobehavioral endpoints [27]. To evaluate the developmental neurotoxicity of TCAB we assessed locomotor activity, and expanded the assessments to include pre-pulse startle inhibition, conditioned avoidance, Morris water maze, and a delayed non-matched to position operant procedure in animals between the ages of PND21 and PND150. The higher level of mortality observed in the 10 mg/kg/day dose group and the terminal assessments at weaning prohibited evaluation of animals as adults due to the diminished number of animals available as assigned to behavioral assessments. Thus, post-weaning behavioral assessments were limited to the control, 0.1, and 1.0mg TCAB/kg/day dose groups.

#### 2.4.1. Locomotor Activity

Between PND37 and 43 rats (n=10) were assessed for exploratory activity in a photocell activity system utilizing animal cage chambers (San Diego Instruments, San Diego, CA). Ambulatory activity was recorded in 5-min epochs over a 60-min test session.

#### 2.4.2. Startle Response and Pre-pulse Startle Inhibition (PPI)

Between PNDs 65-75, rats (n=10/group) were assessed for auditory startle response, habituation, and PPI as a measure of sensorimotor gating using a computer assisted Startle Response System (San Diego Instruments, San Diego, CA). Following a 5-min habituation, under a 70dB background, the 30-min test session consisted of 5 pre-test 120 dB trials followed by a main test unit of 40 trials presented at 15-sec fixed intertrial intervals (ITI) followed by 5 120 dB post-test trials. Main test unit session trials were comprised of no-stimulus, acoustic startle stimulus (40 msec; 120 dB) alone, and pre-pulse stimulus trials (20 msec pre-pulse [3,6, or 12 dB above background: 73, 76 and 82 dB] followed by 100 msec gap and 40 msec pulse) presented pseudo-randomly. 120dB startle amplitude (Vmax) was collected across a 100 msec sampling-window. Data was collected as the mean startle amplitude (Vmax) to 120 dB within the first 5 pre-test trials, the main test session, and the 5 post-test trials. PPI was calculated as a percentage of 120dB startle response by [Vmax of prepulse + 120dB startle/ Vmax of 120dB startle alone] × 100. Data for each averaged value on each kind of trial in the pre-test and post-test, startle alone; in the main test, startle alone, each prepulse intensity alone, startle preceded by each prepulse intensity, and blank were analyzed.

#### 2.4.3. Forelimb and Hindlimb Grip Strength

Between PND70-75, subsequent to the startle assessment, rats (n=10/group) were assessed for grip strength using a 5-kg digital force gauge (Chatillon; AMETEK, Inc., Largo, FL) fitted with a T-bar and attached to a trough platform. Within each trial the rat was assessed for both forelimb and hindlimb strength over 3 consecutive trials. The mean response was calculated for each animal.

#### 2.4.4. Conditioned Avoidance

Between PND75-85, rats (n=10/group) were evaluated for conditioned avoidance response using Coulbourn Instruments, LLC (Allentown, PA) shuttle-boxes placed inside light- and sound-attenuating enclosures. Rats were allowed 2 min to explore both sides of the apparatus. Training initiated with the delivery of a tone and cue light in the chamber containing the rat. Within 5 sec, a shock (0.35 mA, 60Hz) was delivered for 5 sec to the signaled chamber and the animal was required to escape to the adjacent chamber. This process was repeated under a 20-sec variable ITI for a total of 100 trials per session. One training session and two retention sessions were conducted at 24-hr intervals for a total of 3 test sessions over 3 days. Responses were recorded as avoidance (exit during 5-sec cue interval), escape (exit during shock interval), or omit (escape loss with failure to escape during shock interval). The number of crosses between chambers occurring between trials (inter-trial crosses) and avoidance latencies were recorded.

#### 2.4.5. Morris Water Maze

At PND91-95, rats (n=10/group) were assessed for spatial learning and reference memory in the Morris Water Maze (MWM). Rats were randomly placed facing the rim of the pool at one of 5 starting points within a MWM (5’ diameter) and allowed 90 sec to swim and escape to a 4” × 4” submerged platform. Rats remained on the platform for 30 sec prior to removal. Acquisition of the task was conducted with one trial a day for 10 consecutive days. Latency to find the platform and path length were calculated from video recordings (Chromotrack VERSION 4.01, San Diego Instruments, CA). On day 11, a 90-sec probe trial was conducted with removal of the platform. Dwell time and path length in each quadrant were recorded. On days 13-14, 2 days of reversal learning of a different platform location was conducted. Latency and dwell time were confirmed by human observation.

#### 2.4.6. Delayed Non-Matched to Position

At PND110-114, 2 rats per litter per dose group were reduced to 85% free feeding weight over a 12-day period and maintained at that weight throughout the test period. Rats were placed in operant chambers, inside light and sound attenuating enclosures (Coulbourn Instruments, LLC, Allentown, PA). Animals were given 2 daily (days 1-2) autoshaping sessions under a mixed continuous reinforcement (CRF) fixed-time (FT) 1-min schedule for a total of 35-pellets delivery. This was followed by 5 daily sessions (days 3-5; 8-9) under a CRF for 75 reinforcements or 15 min. On days 10-12, rats were trained to press alternating levers. Upon pressing the lever within the 30 sec interval, a pellet was delivered, the lever retracted, and the trial ended. In absence of a bar press, the trial ended after 30 sec. Trials were separated by a 5-sec ITI and the session ended after 75 reinforcements or 15 min. The next daily sessions (days 15-19; 22-25) increased the complexity of the task. Under a CRF with a 20-sec limited hold schedule, one lever was presented. Pressing of the lever delivered a food pellet and the lever withdrawn. Following a 5-sec interval, both levers were presented and the animal was required to press the new lever for reward. A press of the previously reinforced lever would result in lever withdrawal and end of trial in the absence of reinforcement. A 20-sec cut-off was imposed for each trial. Following a 10-sec ITI, the next trial was initiated with a random alternation of levers. The session ended after reaching either 35 choice trials or 30 min. In each session, the number of reinforcements was determined. Lever bias was evaluated and excluded as a factor. In the final 10 sessions, the primary endpoint was accuracy defined as whether after pressing the lever, the animal correctly selected the alternate lever when two were subsequently presented.

#### 2.5. Tissue Collection

At PND21 (n=11/group) and PND120 (n=10/group) underwent necropsy of the peripheral organs (kidney, liver, thyroid gland, thymus, spleen, heart, lungs, testes, and epididymides) following CO_2_. Tissue was excised and absolute organ weights and individual organ weights relative to body weight (relative weight) were recorded. The PND21 rats (n=11/group) were decapitated and the brain excised from the cranium. The forebrain was dissected in the mid-sagittal plane and the left hemisphere immersion fixed in 4% paraformaldehyde/phosphate buffer (PF/PB; pH 7.4) overnight, transferred to cacodylate-buffered saline, then PB saline (1% sodium azide), and stored at 4°C. Brain tissue for histological analysis from PND150 rats (n=10/group) was collected following whole-body perfusion of saline followed by PF/PB under Nembutal^TM^ anesthesia and processed for histology.

### 2.6. Histology and Immunohistochemistry

The characteristic profile of severe developmental hypothyroidism induced by known goitrogens on brain development includes delays in cortical layering, cerebellar granule cell migration, disrupted cerebellar Purkinje cell arborization, altered astrocyte and microglia morphology, and delayed myelination. Thus, we examined brains from PND21 rats for histological endpoints associated with each of these developmental processes in addition to general neuropathology assessment of cell death and injury-induced gliosis. Brain hemispheres (n=11/group) were cryoprotected (20% glycerol:2% dimethylsulfoxide) and embedded in the parasagittal plane in a gelatin matrix representing all groups (MultiBrain^TM^ Technology; NeuroSciences Associates, Knoxville, TX) cured by rapidly freezing in −70°C isopentane. Frozen blocks were cut in a systematic-random manner into 14 sequential 40µm sections separated by 640um intervals. All serial 40µm frozen sections were collected from each animal using a sliding microtome and stored in an antigen preserve solution (50% PBS, PH 7.0; 50% ethylene glycol; 1% polyvinyl pyrrolidone). A systematic random sampling of every 10^th^ section provided sequential adjacent free-floating sections that were histochemically stained for hematoxylin and eosin (H&E), Nissl (thionine), solochrome (myelin tracts), amino cupric silver stain (CuAg; cell degeneration), and immunohistologically stained for ionized calcium binding adaptor molecule 1 (Iba- 1; microglia), glial fibrillary acidic protein (GFAP; astrocytes) in the PND21 rats. In the PND150 rats (n=10/group) H&E and Nissl staining was conducted. Sections mounted on gelatin-coated slides were dehydrated through a graded series of alcohols followed by chloroform/ether/alcohol (1:2:1) and rehydration prior to staining for Nissl with 0.05% thionine/0.08M acetic buffer (pH 4.5) or H&E. For PND21 brains, amino CuAg staining for degeneration was conducted according to published protocol [47]. Solochrome staining for myelin was conducted with incubation of sections with solochrome staining solution (80 ml (1.5% solochrome, 2.5% v/v sulfuric acid), 320 ml dH_2_O, 100 ml 4% ferric ammonium sulfate) for 30 min at RT, rinsed with dH_2_O, differentiated in 1.25% potassium ferricyanide sulfate/0.05% sodium borate decahydrate, rinsed, and counterstained with Neutral red.

For immunostaining, sections from the PND21 rat brains were treated with hydrogen peroxide, blocked with non-immune serum, and incubated in 0.3% Triton X-100 with either rabbit polyclonal antibody to GFAP (astrocytes; 1:20,000; Dako, Carpinteria, CA) or ionized calcium binding adaptor molecule 1 (microglia; Iba-1; 1:15,000; Wako Chemicals, Richmond, VA) overnight at room temperature. Sections were rinsed and incubated with biotinylated goat anti-rabbit secondary antibody and rinsed in Tris buffered saline. The reaction product was visualized with avidin-biotin-HRP complex (Vectastain elite ABC kit, Vector, Burlingame, CA) and diaminobenzidine tetrahydrochloride (DAB). Sections were counterstained with Neutral Red, mounted on gelatinized glass slides, dehydrated in a series of alcohols, cleared in xylene, and coverslipped. Sections were examined under light microscopy for pathology. Pathology examination included a severity grading for neurodegeneration targeted by the amino CuAg degeneration stain as based on a 4-point scale [1- minimal; 2- mild; 3- moderate; 4 – marked]. Evaluation of GFAP and Iba-1 staining was based on density of cells stained in various brain regions as compared to vehicle control and graded for hypertrophy on a 4-point scale. Qualitative assessment was conducted on slides stained for myelin. Qualitative assessments were conducted under low magnification (5x) followed by a high magnification (400x) if necessary to define gross cellular abnormalities. All pathology evaluations were conducted by a board certified pathologist experienced in DNT study evaluations.

Distinct morphological characteristics of immunostained astrocytes and microglia as they relate to development and maturation were examined within the hippocampus and frontal cortex on sections scanned under 20x magnification using an Aperio Scanscope T2 Scanner (Aperio Technologies, Inc., Vista, CA) and viewed using Aperio Imagescope v. 6.25.0.1117. A defined region of interest (ROI) was identified within each area (Suppl. Fig. 1, 2). Within the hippocampus this was within the molecular layer (ML) and within the frontal cortex (FL) it was a region identified anterior to the myelinated tracts. A scoring system for morphology was based on previously published work and reflected the various developmental stages of the maturation of astrocytes and microglia [48-51].

### 2.7. Golgi Analysis

From PND21 (n=11/group) and PND150 rats (n=10/group), the cerebellum was immersion fixed in Golgi-Cox solution (mercuric chloride, potassium dichromate) and maintained in the dark for 30 days. Tissue was embedded in nitrocellulose and 180 mm-sagittal sections collected and staining visualized by ammonium hydroxide. Using coded sections from each cerebellum, 10 randomly selected Purkinje cells in the vermis (e.g., in or close to the mid-sagittal region) and parallel to the focal plane, were evaluated for soma size, dendritic branching area, and density. The perimeter of the dendritic arbor was traced via camera lucida to define the area of the dendritic field and the density of the dendritic branching of the individual cell was assessed as determined by number of branches intersecting the grid points using a superimposed eyepiece square grid graticle. To differentiate large, sparsely branched Purkinje cells from the same total amount of dendritic branching in a smaller, more heavily branched Purkinje cell, branching area and dendritic branch density were integrated into a branching index (PCBI).

### 2.8. Hippocampal Stereology

**Figure 2:**
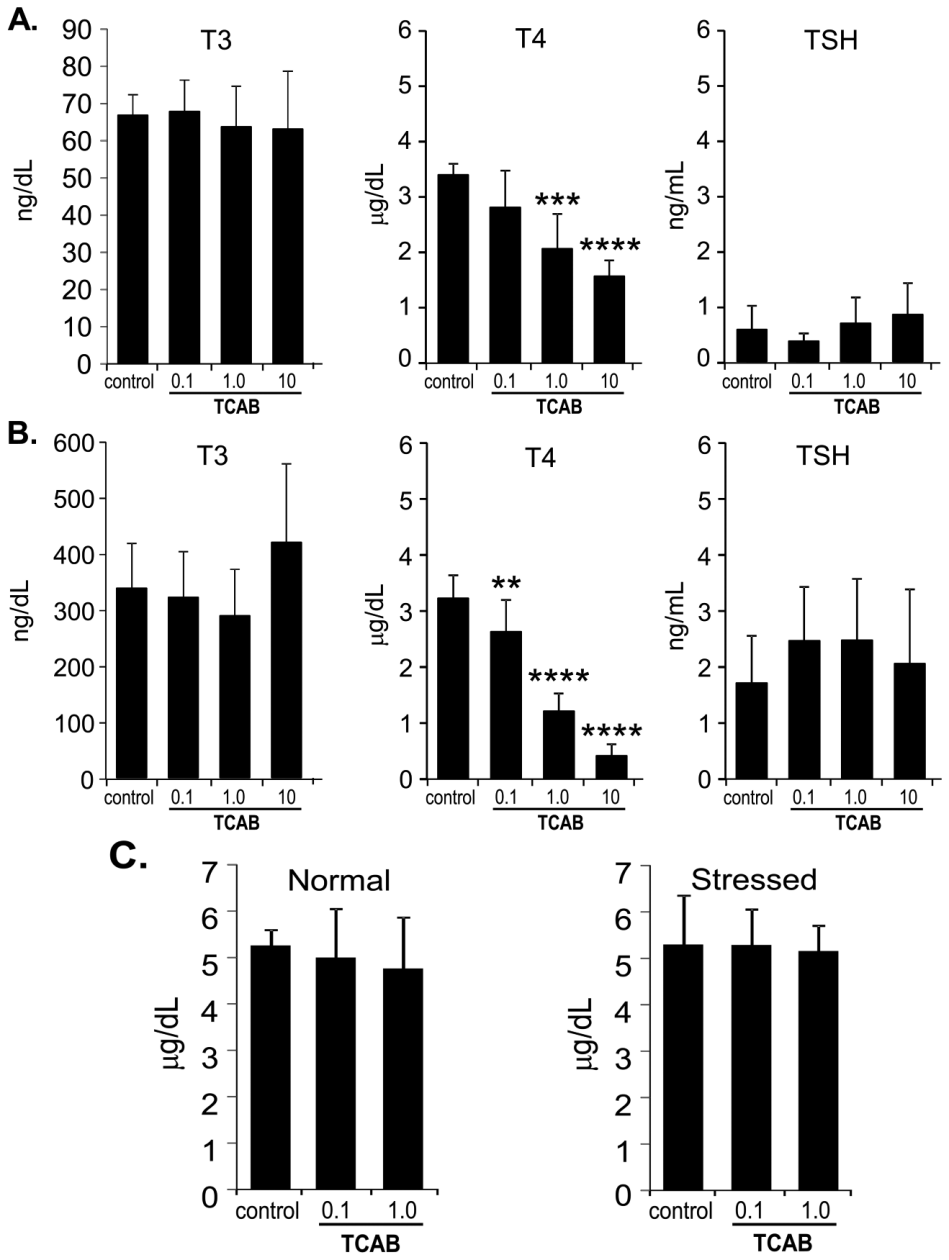
Serum T3, T4, and TSH Levels Serum T3 (ng/dL), T4 (mg/dL), and TSH (ng/mL) levels in (A) dams at PND4 (n=5,5,6,14, respectively for dose) (B) male offspring at PND21 (n=7-9) and (C) T4 levels in adult male offspring (n=10) under normal conditions or following of restraint-stress. Data represents mean+/−SEM. ** p<0.01; ***p<0.001; ****p<0.0001 relative to control.

Computerized stereological analyses (*Stereologer*, Stereology Resource Center, Tampa, FL) of the hippocampal CA1 pyramidal cell (PyC) layer and dentate gyrus (DG) were conducted on thionine-stained sections through the hippocampus of the left hemisphere at PND21 (n=11/group) and PND150 (n=10/group). Under low magnification, reference spaces (DG, CA1) were outlined and the sum of area on the cut surfaces quantified by point counting. Sampling fractions included section sampling fraction (ssf; the number of sections sampled divided by the total number of sections), the area sampling fraction (asf; the area of the sampling frame divided by the area of the x-y sampling step), and the thickness sampling fraction (tsf; the height of the dissector divided by the section thickness). A guard volume of 2 mm was observed above and below the dissector. Using the average post-processing section thickness, the total volume of each reference space for each brain was estimated by the Cavalieri-point counting method [52]. Within each reference space (Fig. 2), neurons were sampled in a systematic-random manner and counted in the x,y,z planes under high resolution, oil immersion, optics (60x, NA 1.4). The total number of neurons (calculated as the product of the sum of neurons counted (∑Q-) and the reciprocal of the sampling fractions) and mean neuron volume (load) were quantified using the optical fractionator and volume fraction methods, respectively [53]. The mean total number of granule cells (GC) in the DG and total number of PyC in the CA1 were estimated using the optical fractionator [54], an unbiased combination of the virtual 3-D probe, the dissector [55]; the fractionator sampling scheme Gundersen’s unbiased counting rules [56], The total number of cells in each region was calculated for each rat as the product of the sum of neurons counted (EQ-) and the reciprocal of the sampling fraction [54, 56].

### 2.9. Statistics

Observational ranked/ordinal data were analyzed by Kruskal-Wallis and binary outcomes using Fisher’s exact test. Organ weights, FL/HL grip strength, ITI crosses in conditioned avoidance (CA), MWM probe trial dwell time and path length, hippocampal stereology measurements, and Golgi measurements were analyzed using ANOVA. Glial scoring was analyzed by Dunnett’s test. Dam and pup serum thyroid hormone levels were analyzed by a one-way ANOVA, a 2x3 ANOVA was used to analyze adult serum thyroid hormone levels with stress. A repeated measures ANOVA was used for body weights, ambulatory activity, startle reactivity, MWM acquisition and reversal learning latency and path length. Latencies within CA sessions were analyzed by ANOVA and a multinomial logit model was fit to the CA data. PPI and Phase I and Phase II of the operant conditioning paradigm were analyzed by a 3x2 ANOVA. A mixed effects logistic regression model for fit for phase III. Independent group means were analyzed by Dunnett’s test or Bonferroni multiple comparison. For all endpoints the dam with litter determined the n size.

## 3. Results and Discussion

### 3.1. Maternal and Pre-weaning Assessments

In the dams, there was no mortality or morbidity and no remarkable exposure-related clinical signs or altered maternal behavior such as nursing (presence of behavior and milk band in pup stomach), nesting, and pup retrieval observed. Two weeks of dosing prior to breeding decreased body weight by 4% [p<0.05] in the 10 mg/kg TCAB dose group and a lower body weight gain continued during gestation of approximately 8% [p<0.05] resulting in a 12% lower body weight in the dams at GD21 [p<0.001] as compared to controls. With cessation of dam dosing no differences in body weight were observed at the time of weaning (post-partum day 21). There were no effects of TCAB on confirmed pregnancies, gestational length, and mean live-litter size of 13-15 pups. Control litters showed a 100% survival until weaning, the 0.1 and 1.0 mg/kg/day groups showed >90% survival while, the direct gavage of the 10 mg/kg/day dose group resulted in a loss of approximately 50% of the pups primarily between PND 14 and 21. Body weight of the pups was not significantly altered by TCAB exposure until after PND14. The decreased body weight gain observed over the 3^rd^ post-natal week resulted in significantly lower weights at PND21 [F(3,38)=22.23; p<0.0001] of 10% in the 1.0 (p<0.01) and 25% in the 10 mg/kg/day [p<0.001] groups as compared to controls. In the 10 mg/kg/day group the body weight deficit was not observed in all animals but rather half of the pups displayed body weights within the low-range of controls. Growth retardation and survival has been previously demonstrated in hypothyroid mice born from dams treated with methimazole/KClO4 with a 90% decrease in serum T4 levels and 50% decrease in T3 [57]. With the cessation of dosing at weaning, no significant differences in body weights were observed at PND28 or later in the 0.1 or 1.0mg/kg/day groups (data not shown). Similar to previous findings for developmental TCAB exposure [32] the initiation of incisor eruption was found to occur an average of 1-day earlier in the 1 and 10 mg/kg/day groups (PND10) as compared to controls (PND11) and eye opening (either eye open) occurred earlier in the 10 mg/kg/day group (PND13) as compared to all other groups (PND14).

Relative weights (g) of right kidney (control: 0.64 +/−0.01; 10 mg/kg/day: 0.83+/−0.03), liver (control 4.52+/−0.09; 10 mg/kg/day: 6.07+/−0.41), and heart (control: 0.56+/10.01; 10 mg/kg/day: 0.97+/−0.09) were increased in the 10 mg/kg/day group [p<0.01] with an associated increase incidence of jaundice. In the current study, relative thymus weight (g) was decreased in all developmentally TCAB-exposed groups (p<0.05; control: 0.42 +/−0.02; 0.1 mg/kg/day: 0.37+/−0.02; 1.0mg/kg/day: 0.30+/−0.01; 10 mg/kg/day: 0.20+/− 0.02) and in adult exposure studies thymic atrophy has been reported following TCAB exposure [58]. Dosing ceased at weaning and no TCAB-related differences were observed in organ weights in the adult. Relative thyroid weights were not altered and there was no evidence of thyroid gland histopathology under the developmental exposure paradigm; however, chronic exposure to TCAB has been reported to produce thyroid gland tumors in male rats [35].

### 3.2. Serum TH Levels

Based on previous reports of hypothyroxenemia induced by TCAB exposure [32-34], serum TH levels were assayed independently for dams, PND21 pups, and adult offspring. During periods of dosing, T4 levels were lower in dams and pups and in the adult offspring T4 levels were no longer diminished. At PND4, maternal serum T4 levels were significantly decreased with TCAB exposure [F(3,26)=25.39; p<0.0001] with significantly lower levels in the 1.0 [p<0.001] and 10 mg/kg/day [p<0.0001] groups (Fig. 2A). In PND21 pups, serum T4 levels were significantly decreased with TCAB exposure [F(3,33)=83.68; p<0.0001] with a significant decrease observed in the 0.1 [p<0.01], 1.0 and 10 mg/kg/day [p<0.0001; 60% and 80%, respectively] pups, as compared to controls (Fig. 2B). No changes were seen in T3 and TSH (Fig. 2A,B). At PND150, serum T4 levels in all dose groups were within range of control values (Fig. 2C).

The observed TCAB-induced deficit in serum T4 levels, in the absence of changes in T3 or TSH, is consistent with previous reports on the effects of TCAB [32, 34]. In those previous studies, T4 glucuronidation as a result of uridine diphosphate glucuronyltransferase induction was considered as a primary mechanism underlying the T4 deficit rather than a direct effect upon hormone production. A similar effect has been reported for other dioxin-like compounds resulting in increased T4 metabolism and clearance [59-60]. In a 3-month oral TCAB exposure study, significant inductions of hepatic 7- ethoxyresorufin-O-deethylase (EROD), acetanilide-4-hydroxylase, and 7-pentoxyresorufin-O- deethylase activities were observed [35]. In the current study, we did not measure hepatic enzyme activity; however, the 25% increase in relative liver weight with TCAB exposure could be associated with an increased hepatic metabolic activity similar to that seen in the previous study.

### 3.3. Neurobehavioral Assessments

A number of behavioral assessments were conducted from the time of weaning into adulthood. At weaning, non-moribund pups randomly selected from all dose groups were examined by the FOB. For all other post-weaning behavioral endpoints, the high dose group (10 mg/kg/day) was excluded due to the general toxicity observed in a portion of the dose group that could confound neurotoxicity assessments and the diminished n size following terminal tissue collection at weaning. At TCAB doses that produced between 15% and 60% reduction in serum T4 during development, no effects were observed in activity, grip strength, startle and pre-pulse startle inhibition and learning and memory tasks.

#### 3.3.1. Functional Observational Battery

Evaluation of sensory and motor functions by an FOB at PND20 identified, a lower activity score (control: 4.4 +/− 0.2; 10 mg/kg: 2.7 +/− 0.3; p<0.05) and hindlimb grip-strength (kg; control: 0.062+/− 0.008; 10 mg/kg: 0.035+/−0.004; p<0.01) that was consistent with the observed lower body weight. While clinical observations and organ histopathology indicated signs of general toxicity at this dose level, no other effects were detected with the FOB assessment. In the two lower dose groups, there were no clinical signs of toxicity, less than 10% decrease in body weight, and the FOB did not detect sensory or motor alterations.

#### 3.3.2. Locomotor Activity and Grip Strength

**Figure 3:**
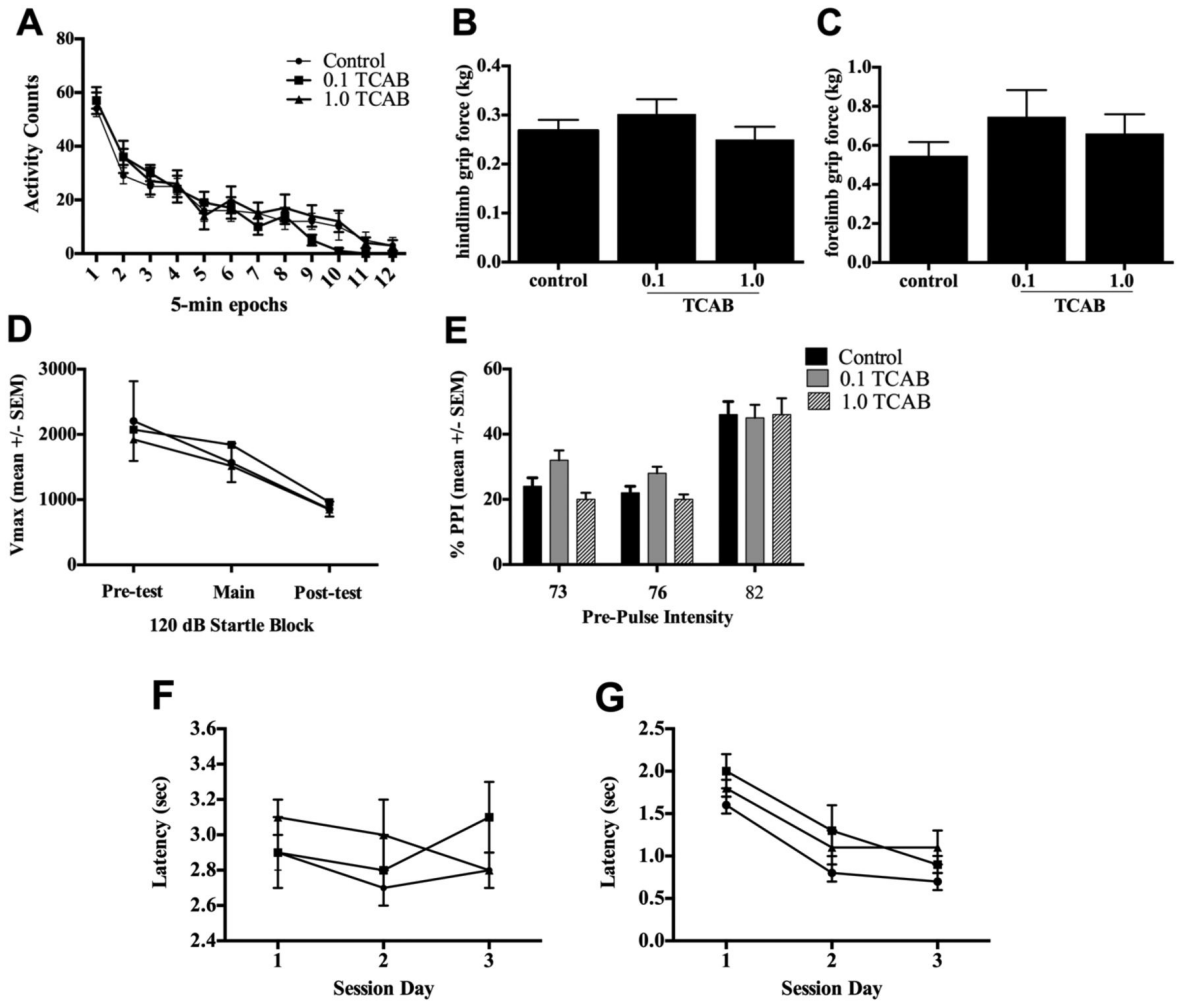
Neurobehavioral Assessments (A) Ambulatory activity in PND 37-43 rats assessed in 5-min epochs over a 60-min session. Data represents mean+/−SEM. (B) Forelimb and (C) hindlimb grip strength. Data represents mean of 3 trials +/− SD. (D-E) Startle response and pre-pulse startle inhibition (PPI) over a 30-min test session (D) 120 dB startle response amplitude (Vmax; 100 msec sampling window). Data represents mean over 5 trials (+/−SEM). (E) Pre-pulse startle inhibition (% PPI). Data represent the mean % inhibition ± SEM. (F-G) Avoidance latency for (F) avoidances and (G) escapes over 3 day sessions. Data represents mean ± SEM. n=10.

At PND37-43, ambulatory activity was not altered by developmental exposure to TCAB. Total ambulatory activity for the 60-min test session was not altered by TCAB exposure. A significant main effect of time was demonstrated [p<0.001] with no effect observed for dose or dose × time interaction (Fig. 3A). Acclimation to the novel environment as measured by a change in activity from the first to the last 5-min epoch was not altered by TCAB exposure. Forelimb and hindlimb grip strengths, as measured in young adults (PND70-74), were not altered by TCAB exposure (Fig. 3B,C).

#### 3.3.3. Pre-pulse Startle Inhibition

In young adults, startle amplitude to 120 dB stimulus (Fig. 3D) did not differ between controls and TCAB dosed rats. The decrease in Vmax over three sessions [F(_2,60_) =15.94, p<0.0001] indicated 50% habituation with no difference observed across dose groups. Each of the pre-pulse stimuli was not significantly different than the blank trials and there was no difference between groups on the blank trial recordings. Pre-pulse startle inhibition (% PPI) was calculated as a percentage of 120dB startle response by [Vmax of prepulse + 120dB startle/ Vmax of 120dB startle alone]x100. A two-way ANOVA (exposure and prepulse intensity as factors) showed an effect of prepulse intensity on startle response [F(_3,120_)=5.97; p<0.05) with no significant effect of exposure and no significant interaction between pre-pulse intensity and exposure (Fig. 3E)*.*

#### 3.3.4. Conditioned Avoidance

**Figure 4:**
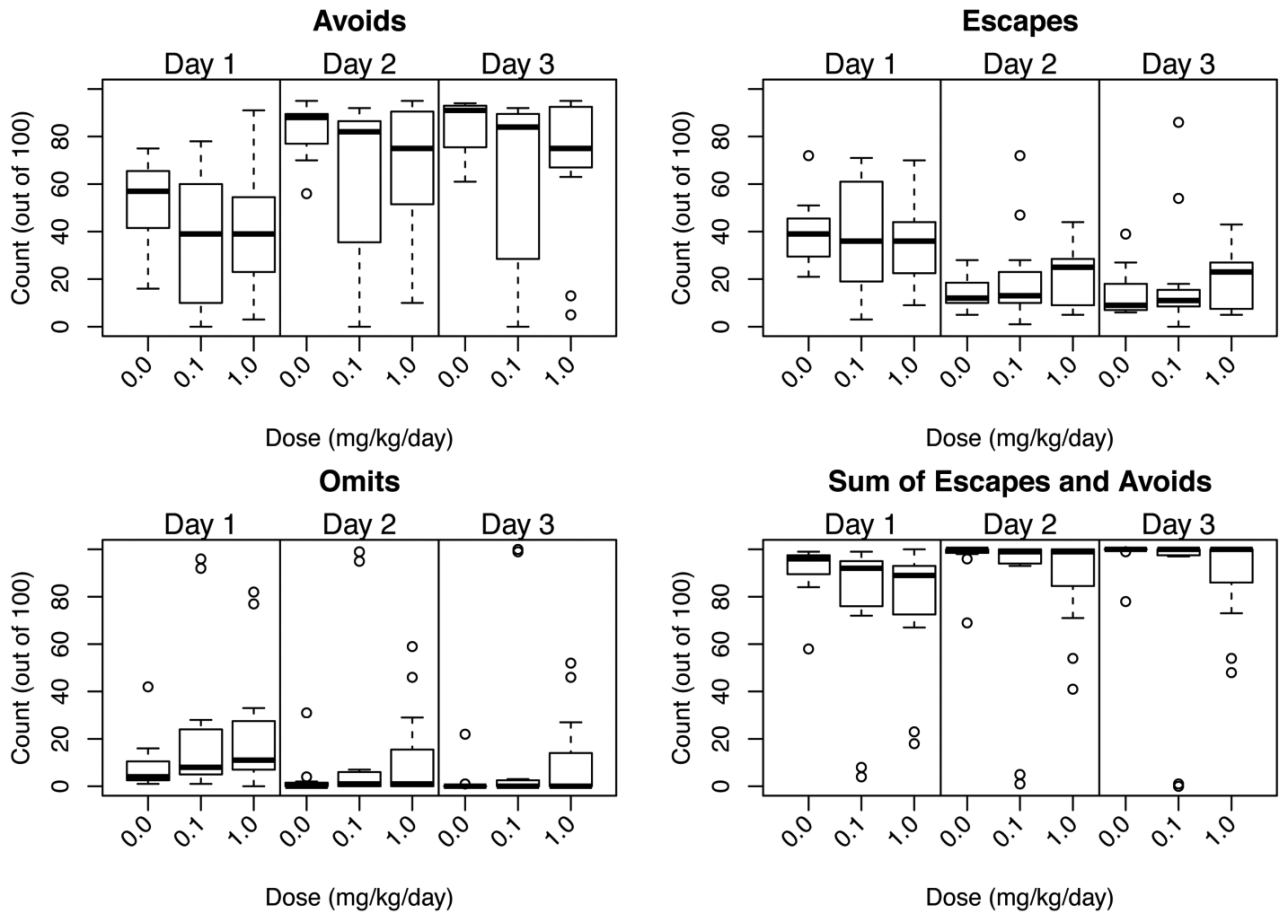
Conditioned Avoidance Boxplot of Avoids, Escapes, Omits and combined Escape/Omit responses over day 1 of training and days 2 and 3 of retention (median number of responses in 100 trials; the upper and lower quartiles of the response distribution; range of distribution, and extreme observations (circles).

Over the progression of the training session, animals learn to associate the light/tone cue with the delivery of a foot shock. The initial response is one of escape upon shock delivery. As the association is learned, this shifts to a more rapid response where the animal avoids receiving a shock (avoidance). If the animal does not learn the association or adopts a freezing response, a loss of response is recorded (escape loss). Responses were recorded as avoidance (exit during 5-sec cue interval), escape (exit during shock interval), or omit (escape loss with failure to escape during shock interval). The number of crossings between chambers occurring during the ITI was not significantly different between controls and TCAB dosed animals indicating similar activity levels and locomotor capability. During the first training session, a similar number of escapes occurred at similar latency for all animals (Fig. 3F) and, within that session, avoidance latency was not significantly altered by TCAB exposure (Fig. 3G). Escape latency did not change over the 3 sessions (Fig. 3F). Avoidance latency showed a general decreased over the sessions with no effect of TCAB exposure. Given the limited response distribution on any one trial, a multinomial logit model was fit to the second day as a function of dose allowing estimation of the effect of a change in dose on the odds of observing an escape relative to avoid, or omit relative to avoid. After accounting for over-dispersion using a heterogeneity factor [61], TCAB exposure was not found to significantly alter response (Fig. 4).

#### 3.3.5. Morris Water Maze

**Figure 5:**
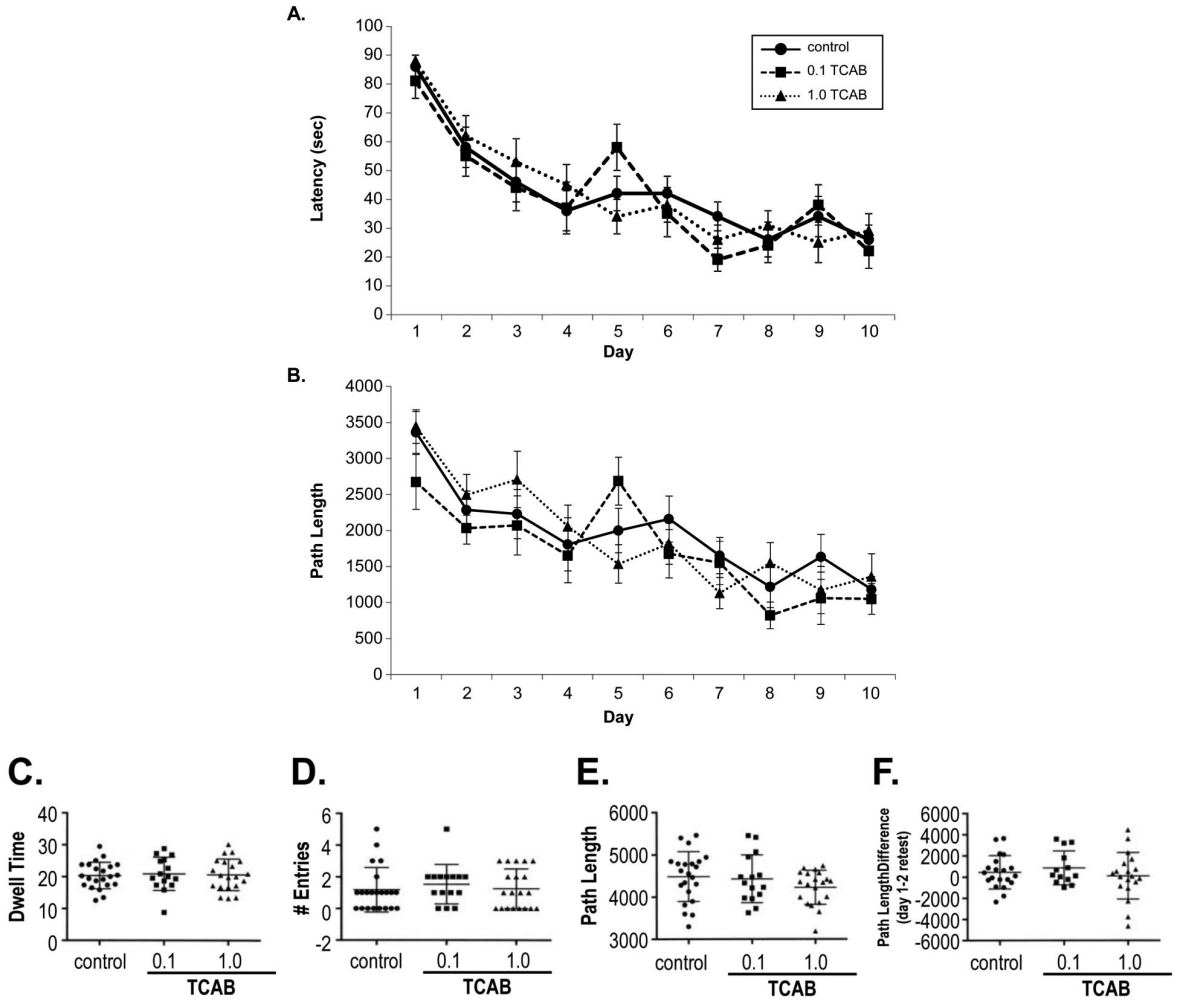
Morris Water Maze Acquisition of a Morris water maze task over 10 consecutive days of training in PND91-95. (A) Latency (sec) (B) path length to reach the hidden escape platform. In the probe trial (C) dwell time (sec) within the platform quadrant, (D) number of entries into the platform quadrant, and (E) path length were recorded. (F-G) Reversal learning (F) latency and (G) path length. Data represents mean +/− SEM, n=10.

Total acquisition, as assessed by latency to find the hidden platform during the ten days of training, was not significantly altered by TCAB exposure. No differences were observed for swimming distance or in swimming velocity during the test sessions (data not shown). All animals demonstrated acquisition in latency [Fig. 5A; F_(9,486)_=28.0; p=0.0001] and path length [Fig. 5B; F_(9,486)_=18.72; p=0.0001] with no differences observed as a function of TCAB exposure. On day 11, a 90-sec probe trial was conducted with removal of the platform. Dwell time within the platform quadrant was similar between all dose groups (Fig. 5C). Time spent in the remaining quadrants (Fig. 5D) and path length (Fig. 5E) showed a similar preference pattern across all groups. When rats were tested for the ability to shift their response to a new quadrant, reversal learning, all animals showed a significantly shorten latency on the first day than that observed in the initial training [F_(1,54)_=4.38; p=0.04], returning to within previous performance latencies by the 2^nd^ day. No significant differences were observed between the TCAB treated rats and controls (Fig. 5F).

#### 3.3.6. Delayed Non-matched to Position

**Figure 6:**
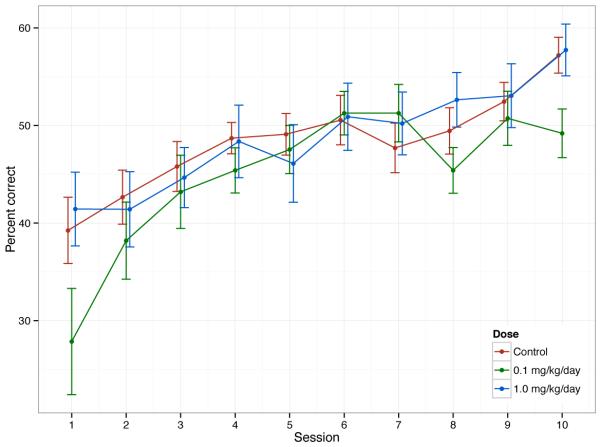
**Delayed Non-Matched to Sample** in Phase IV over 10 Days. Data represents mean +/− SD.

Working memory has been empirically defined as a short-term memory for an object, stimulus, or location used within a testing session [62]. Behavior within a task is guided by a delay-dependent representation of stimuli. An example of a delayed response working memory task is the delayed non- matching to position task developed by Dunnett [63]. In this task the level to which the rat responds serves as the to-be-remembered stimulus. This method has been used effectively to demonstrate hippocampal and extrahippocampal alterations [64]. Two animals from each individual litter were used to ensure availability of animals trained to criteria prior to each phase of testing. In the initial autoshaping training for bar pressing all animals demonstrated the ability to learn and perform the task with all animals reaching criteria of an 80% success rate (Phase I). With the further training of the animals on a CRF schedule (Phase II), all animals successfully performed that task reaching criteria performance at the end of Phase II. In Phase III, the animals were presented a delayed memory schedule for 3 days. For each day, a mixed effects logistic regression model was fit for the number of reinforcements as a function of dose, with a random effect to represent the nesting of rat within litter. The log odds of seeking reinforcements during a 15-min period were modeled as a function of dose with additional variance components estimated to account for possible clustering in response by litter. There was no significant effect of TCAB exposure on performance in Phase III. In Phase IV acquisition of performance on the delayed-memory schedule over 10 days was assessed. Using an optimal 5-sec delay interval, previous reports in the literature have indicated a gradual increase in percent correct over the first 4 100-trial sessions (shifting from 50% to 60%), reaching an average of 85% correct following 11 sessions [65]. In the current study, all groups demonstrated an average of 40% correct at the beginning of Phase IV increasing to >50% on day 10 (Fig. 6). A repeated measures ANOVA was fit to the percentage correct as a function of session number and dose group. Model errors followed a lag 1 temporal autocorrelation structure; that is, the correlation between two observations decreased in absolute value with time between sessions. There was a significant effect of session number (p<0.0001) but no significant effect of dose or dose by day interaction.

### 3.4. Brain Histology and Immunohistochemistry

At PND21, neuropathology evaluations were conducted on all brain sections collected for unbiased stereology. In addition, as specific anatomical features known to be affected by developmental hypothyroidism, we examined cortical and cerebellar layering, myelin, microglia and astrocyte morphology. At PND21, histological analysis of the brain was conducted for all dose groups. Nissl and CuAg staining showed no evidence of cell death or lesion sites across brain regions as evaluated by conventional neuropathological assessments. During normal development, the process of synapse pruning (removal of excess synapses) can be detected by CuAg staining. In the hippocampus, this was evident within the mossy fiber bouton region as the synaptic field of the dentate granule neurons. Qualitatively, the controls and the 0.1 and 1.0 mg/kg/day animals displayed similar patterns while, at the higher 10 mg/kg/day dose, this distinct staining pattern was qualitatively less prominent, suggestive of less requirement for synaptic pruning (data not shown). While this was an interesting observation and would be consistent with other effects observed on dentate granule neurons, further studies are necessary to confirm this qualitative observation.

#### 3.4.1. Minimal Effect of TCAB on Cortical Layering

**Figure 7:**
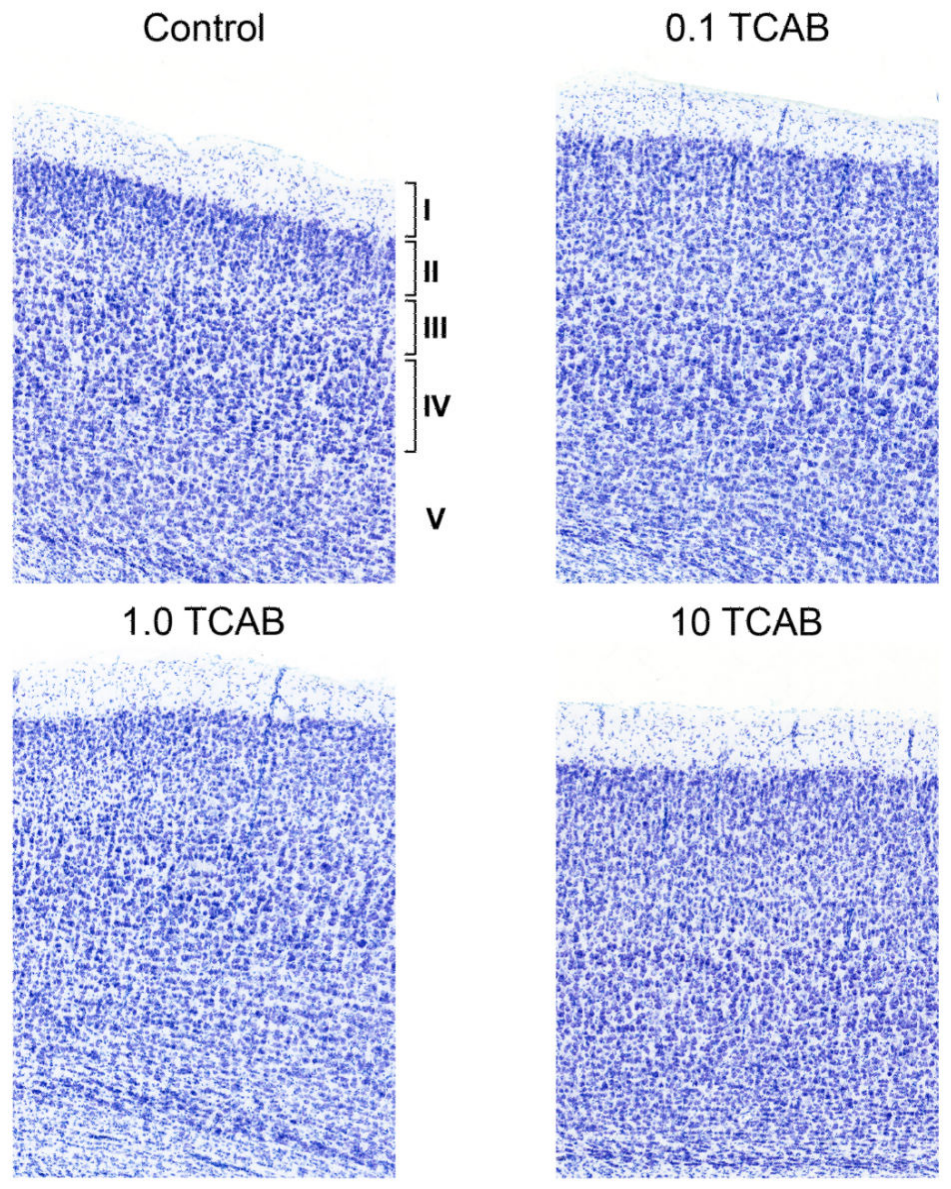
Representative Nissl staining of the cortex at PND21 Cortical layers I, II-III, and IV were identified in the controls and 0.1 mg/kg/day TCAB dosed animals while layer V was more diffuse. The 1.0mg/kg/day group showed a less-well compacted layer IV with diffusion into layer III. In the 10 mg/kg/day group, the distinction between layers II-III and IV was not well defined. No differences were observed in adults (data not shown).

The cortex is organized into layers that are established with the migration of neurons along radial glia. We observed distinct layers I, II-III, and IV in all control and 0.1 mg/kg/day rats (Fig. 7). In all animals layers V-VI were more diffuse, consistent with the developmental stage of cortical development. This was observed upon visual inspection and confirmed by ranking (presence/absence) by experimenters, blinded to dosing assignment, with regards to identification of each layer across 3 stained sections of the cortex for each animal of all dose groups. A clear demarcation of layers V-VI was identified in only 3 of the total brains examined in the study which is consistent with the ongoing maturation and layer compaction of the cortex at PND21. In the 10 mg/kg/day rats, a distinction between layers II-III and IV was qualitatively not as well defined as in the controls and the 1.0 mg/kg/day group. This visual observation was confirmed by a similar ranking approach and only 40% of the animals in the 10 mg/kg/day group displayed a clear demarcation between layers II-III and IV.

#### 3.4.3. Absence of Anatomical Effects of TCAB on Myelin

**Figure 8:**
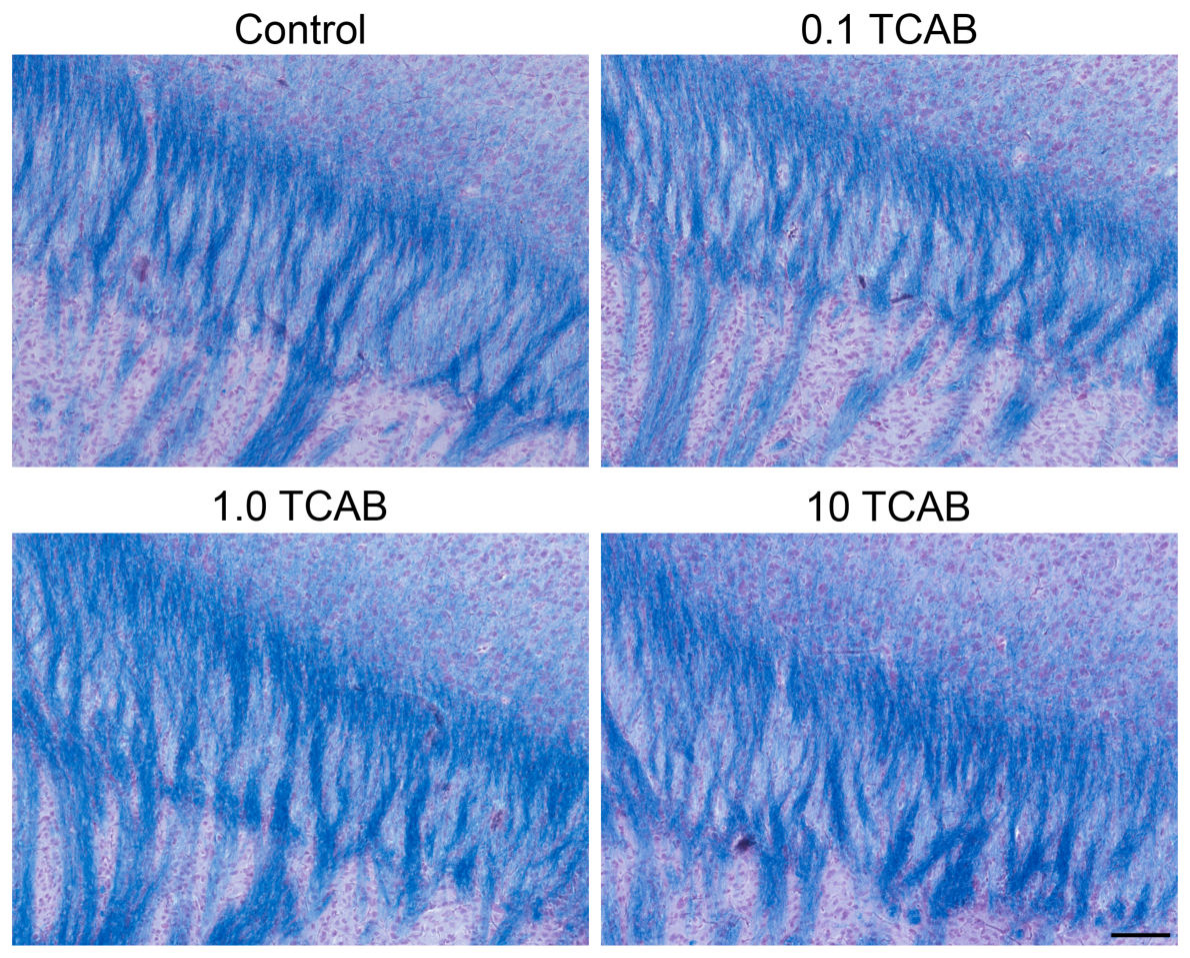
Myelin. Representative solochrome staining of myelin (blue) in the corpus callosum at PND21. A normal pattern and density of staining with no visual differences observed as a function of developmental TCAB exposure. Scale bar = 50µm.

TH regulates differentiation of oligodendrocytes and myelination [37, 66-73]. Deficits in TH have been reported to diminish oligodendrocyte development [68, 69] and accumulation [71], as well as, delay myelin initiation [72-73]. In addition, a delay in the expression of genes encoding structural proteins of myelin [72] has been reported under TH deficits from the first postnatal week to 30 days of life [70, 73]. At PND21, staining of myelin in the corpus callosum and of projections into the cortex appeared relatively uniform across groups with no evidence of hypomyelination or dysmyelination based upon the conventional neuropathological evaluation (Fig. 8). This would suggest a normal progression of myelin accumulation; however, as we did not examine the anatomy or myelin-related markers at an early age we cannot exclude the possibility of an earlier effect on oligodendrocyte development or myelin initiation that may be the more sensitive indicators of TH disruption [68-73]. Previous work has reported the presence of an aberrant cluster of neurons termed heterotopia in the corpus callosum in a model of developmental T4 deficit via maternal low dose PTU [74]. Conventional histopathological examination of the tissue did not report heterotopia in the myelinated tracts of corpus callosum in TCAB exposed animals was not reported in the conventional examination of the tissue.

#### 3.4.4. Morphological Features of Microglia and Astrocytes Related to Development rather than Gliosis

One rationale for examining a morphological change in astrocytes or microglia within a neurotoxicity evaluation is based upon the anatomical hypertrophy that occurs in these cells in response to injury [75-76]. However, glial cells also demonstrate anatomical changes as they mature over the course of brain development that differs from the hypertrophy seen with injury. These maturational changes reflect the changing demands of the brain over the course of post-natal development. As such considering these phenotypic features is relevant to the neuroanatomical evaluation in any developmental neurotoxicity study. In rodents, microglia transition over the first postnatal month from an amoeboid to a process bearing morphology and the complexity of processes continues with maturation [50, 77-78]. These morphological changes have been related to various functions of the cells during brain development [79-81]. In a similar fashion, over the course of brain development, astrocytes display a maturational progression characterized by cell body enlargement and increase in process complexity and thickness [82-83]. This maturation progression is also associated with various functions of the cells and production of neurotrophic factors [84]. Astrocytes are responsive to TH with evidence that TH influences their differentiation and maturation of full processes [41, 85-87]. Microglia also display responsiveness to TH and are likely to express TH transporters [88]. A maturational delay of microglia is induced by hypothyroidism as characterized by a significant decrease in the number of process-bearing cells and with hyperthyroidism, microglia development is accelerated [42, 89].

Based upon the severity rating scale of neuropathology outlined in the methods section, astrocyte hypertrophy and microglia activation that are normally associated with brain injury and cell death, were not observed in any of the TCAB dosed animals. This lack of gliosis would be consistent with the absence on any evidence of neuronal death or pathology. However, using a method allowing us to score the morphological phenotype of the cells, TCAB exposure-related effects were identified suggestive of a developmental delay in process formation.

##### 3.4.4.1. Astrocytes

**Figure 9:**
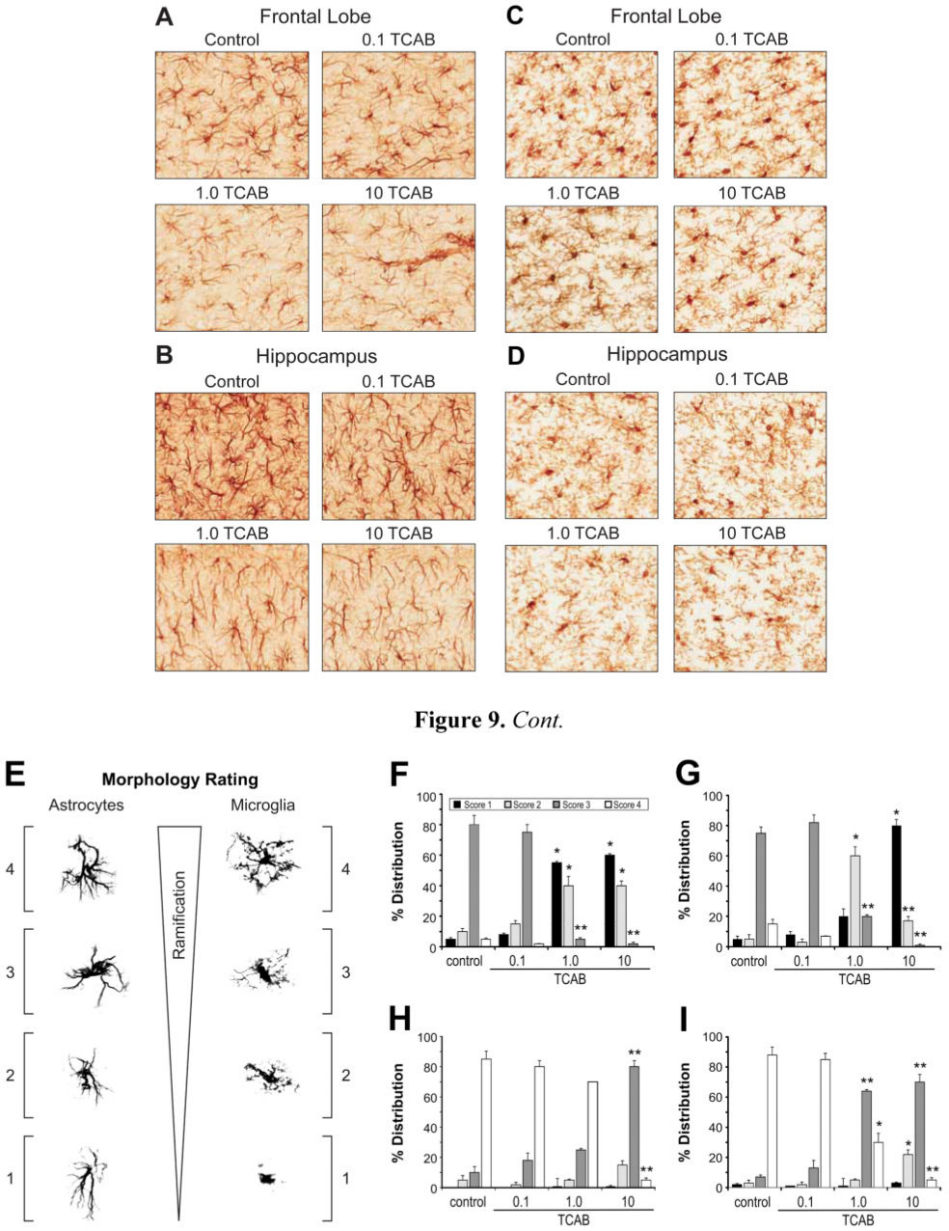
Astrocytes and Microglia Response to TCAB. Representative image of GFAP+ astrocytes and Iba-1+ microglia (DAB brown staining) at PND21 within the (A,C) frontal lobe and (B,D) hippocampus of control and 0.1, 1.0, and 10 mg/kg/day TCAB developmentally exposed rats. Astrocytes displayed distinct cell soma and densely stained processes in controls and the 0.1 mg/kg/day groups. In the 1.0 and 10 mg/kg/day groups, cells displayed thin processes and less branching complexity. Microglia in the control brain exhibited complex and dense process extensions. These processes were less complex in the 10 mg/kg/day dose group resulting in diminished immunoreactivity product between cell bodies. Scale bar [---] = 25µm. (E) Ranking of glia morphology on the basis of cell soma shape, orientation of processes, density of processes, and complexity of processes. Percentage of each stage for astrocytes in the (F) FL and (G) ML and microglia in the (H) FL and (I) ML. Data represents mean +/− SD. *p<0.05; **p<0.01 as compared to control.

A relatively uniform distribution of GFAP+ astrocytes was observed throughout the frontal lobe (FL) and hippocampal ML (Fig. 9A,B;) of all animals with no evidence of hypertrophy. In the FL, GFAP staining of distinct dark cell bodies and processes was similar between controls and the 0.1 mg/kg/day group (Fig. 9A). At 1 and 10 mg/kg/day, decreased immunoreactivity was observed with astrocytes showing less distinct cell bodies and diminished thickness and complexity of processes as compared to controls (Fig. 9A). In the hippocampus, control and 0.1 mg/kg/day animals displayed similar morphologies for GFAP+ astrocytes characterized by a dense cell body and distinct processes. This morphology was altered in the 1 and 10 mg/kg/day groups with the cells displaying less distinct cell bodies and thinner processes (Fig. 9B). Using a modified rating scale to quantitate the distribution of cells within each morphological phenotype (Fig. 9E), the mean percent distribution of cells at each score, relative to the total number of cells within a defined ROI, was determined from two sections matched to plane of cut from each animal. This rating demonstrated a significant shift in the prominent morphological phenotype for GFAP astrocytes dependent upon TCAB exposure (Fig. 9F,G). In the FL, a prominent distribution of #3 GFAP+ astrocytes was observed in the controls and 0.1 mg/kg/day group (Fig. 9F). In the 1.0 and 10 mg/kg/day groups this profile shifted with a significantly lower percentage of astrocytes scoring at #3 [p<0.01], as compared to controls. In addition, a significantly greater percentage of astrocytes scoring at #1 or #2 was observed in both the 1.0 and 10 mg/kg/day groups [p<0.05], as compared to controls. In the hippocampus, the controls and 0.1 mg/kg/day group showed a predominance of cells scoring #3 (Fig. 9G). A shift in this phenotype profile was observed in the 1.0mg/kg/day group characterized by a significant decrease in #3 scored astrocytes [p<0.01] and a significant increase in cells scoring #2 [p<0.01], as compared to controls. At the 10 mg/kg/day dose level, the phenotype profile was characterized by significant increase in the percentage of cell scoring #1 [p<0.01] and #2 [p<0.01] and a significant decrease in cells displaying a score of #3 [p<0.01], as compared to controls.

Astrocytes are the primary neural cell expressing type 2 iodothyronine deiodinase (D2) in the neonatal rat brain [12]. It is thought that astrocyte D2 serves in a manner to provide T3 for neurons that express thyroid receptors but lack T3 protein synthesis capability [90-91]. In the adult, and likely the neonate, rapid adaptation to hypothyroidism to maintain T3 levels in the brain within a normal range occurs via increased astrocyte D2 and decreased neuronal D3 to increase T3 generation and reduce T3 degradation. The efficiency of this adaptive response in the fetal brain is not understood but has been speculated to be associated with glial maturation as the intracellular machinery allowing astrocytes to respond to T3 results in secretion of growth factors necessary for regulation of extracellular matrix protein secretion and organization and neurite growth and survival [84, 92-95]. T4 can have direct effects on the organization of F-actin filaments in astrocytes to promote integrin clustering and focal contact formation [96-98], each of which are fundamental in the regulation of developmental neural cell migration. It is possible that the morphological differences in astrocytes observed following developmental TCAB exposure reflect increase demands placed on these cells at a time when they are required to provide a high level of growth factors for brain development. Whether the changes are dependent or independent of neuronal effects, a delay in the normal maturation of glia could have significant impacts on developing neurons.

##### 3.4.4.2. Microglia

Iba-1 staining for microglia showed a relatively uniform distribution of cells in both the FL and the hippocampus with no evidence of activation normally associated with brain injury (Fig. 9C,D). Microglia soma size (approx. 1-3 µm) was not altered and measurement of distance to “nearest neighbor” indicated a normal spacing (approximately 52 µm) between cells. The absence of immunoreactivity between cell bodies suggested diminished microglial processes. Microglia with ramified-processes extending in multiple directions were seen in controls and at 0.1 mg/kg/day. In animals receiving 1.0mg/kg/day, microglia displayed more of a rod-like morphology with less processes. At 10 mg/kg/day, microglia displayed elongated lumpy cell bodies with processes extending in a primary direction rather than the highly ramified cells observed in controls. To quantitate these differences in microglia phenotype, all cells within each ROI were scored for their morphological phenotype (Fig. 9E). The mean percent distribution of cells within each score was determined for each ROI in two sections matched to plane of cut from each animal. In the hippocampus ML and the FL, microglia morphology scored predominantly at #4 in controls and 0.1 mg/kg/day group. In the FL there was a slight shift with an increase in cells scoring #3 in the 1.0mg/kg/day group that failed to reach statistical significance (Fig. 9H). In the 10 mg/kg/day group the predominant score for microglia in the FL was #3 that was significantly elevated [p<0.01] over the percentage of #3 cells observed in controls. The percentage of #4 microglia was significant decreased [p<0.01] in the 10 mg/kg/day rats as compared to controls. In the hippocampus a difference in the percentage of microglia scoring at #3 or #4 was observed in both the 1 and 10 mg/kg/day groups (Fig. 9I). Significantly fewer cells scoring #4 [p<0.05; 0.01, respectively] and a greater percentage of microglia scored at #3 [p<0.01] as compared to controls. In the 10 mg/kg/day group, a significant increase was also observed in the percentage of microglia scoring #2 [p<0.05], as compared to controls (Fig. 9I).

### 3.5. Computerized Stereology Of Hippocampus

**Figure 10:**
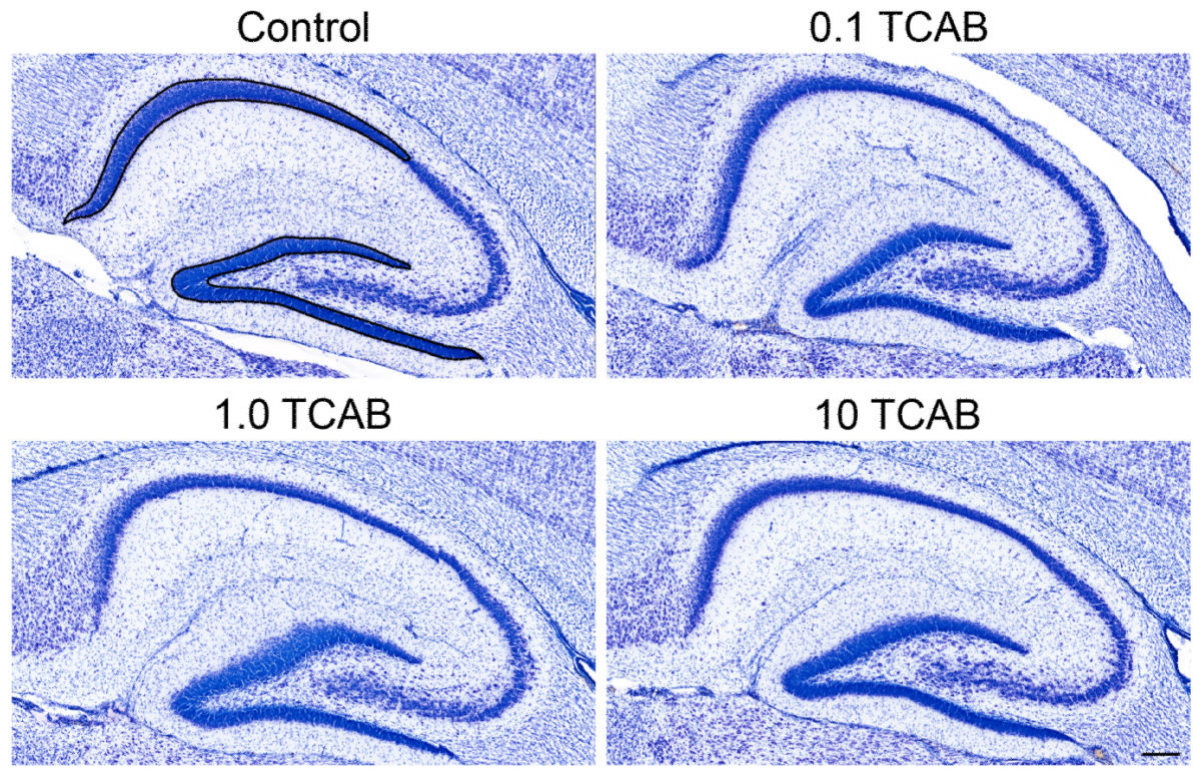
Representative thionine-stained sections through the hippocampus at PND21. No major effects were observed on the structure of the hippocampus following TCAB. Reference spaces for stereology assessment of the hippocampal dentate gyrus (DG) and CA1 pyramidal cell (PyC) regions are outlined in dark lines in the control image. Scale bar 100 µm.

Alterations in hippocampal development attributed to T4 deficits with hypothyroidism include reductions in dentate granule neurons [38] and CA pyramidal neurons [39]. Following TCAB and the associated T4 deficit, we found no overt changes in the hippocampal morphology; however, the use of unbiased stereology detected deficits in neuronal number within defined regions (Fig. 10).

**Figure 11:**
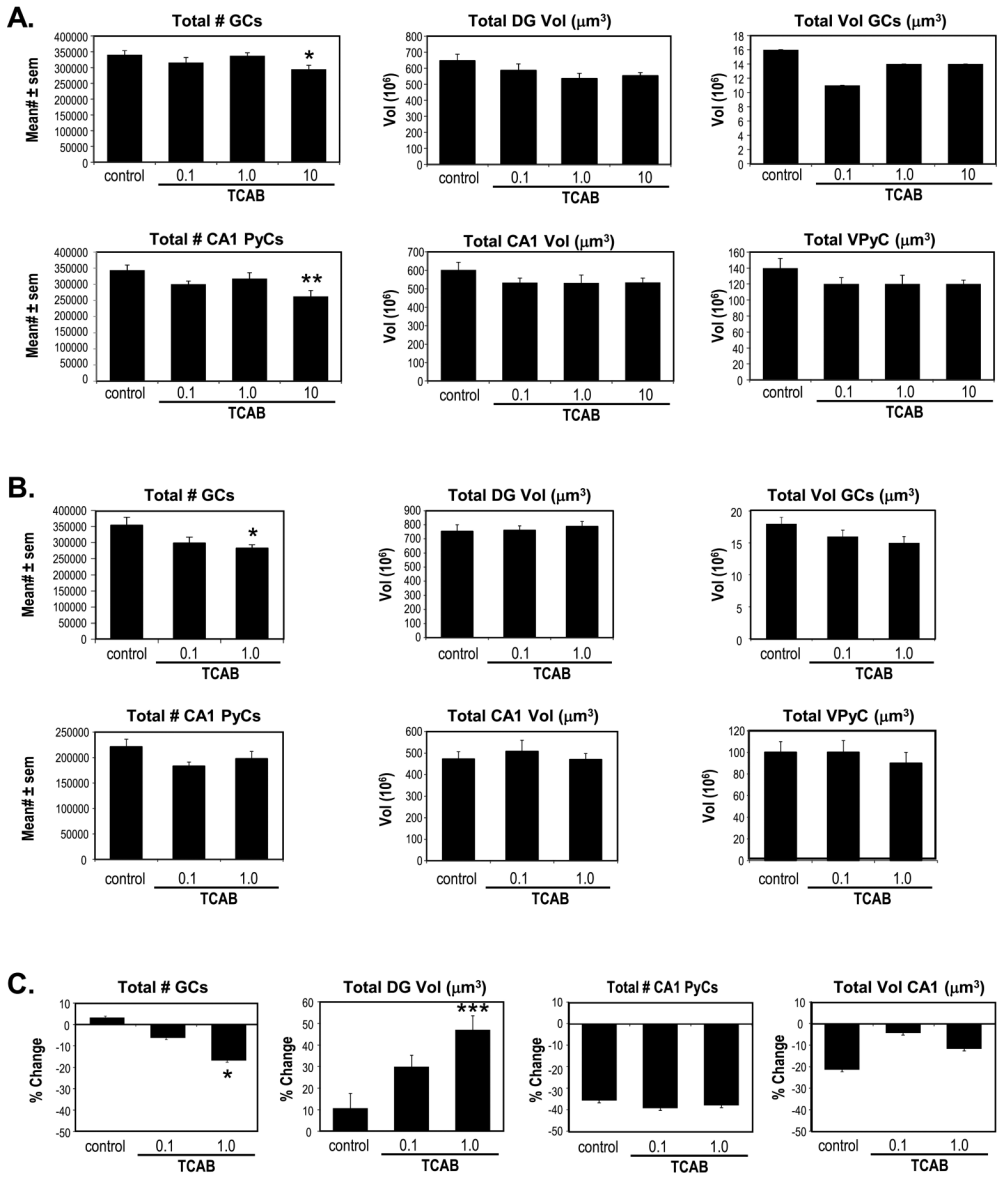
Hippocampal Stereology Unbiased stereology of hippocampal dentate granule cell region (DG) and CA1 pyramidal region in (A) PND21 and (B) PND150 male rats following developmental exposure to TCAB. Total number of dentate granule cells (GC) and CA1 pyramidal cells (PYCs), regions of the DG and CA1, and total volume of the GC and CA1 pyramidal cells (VPyC) were calculated. (C) % change as a function of maturation from PND21 to PND150 for each endpoint. Data represents mean +/− SEM. *p<0.05; **p<0.01; ***p<0.001 as compared to control.

At PND21 the hippocampus is continuing to form and, with ongoing cell migration and the absence of an adult level of compaction, the different DG and CA1 regions remain relatively diffuse, which affected the overall volume measurements. The features of the hippocampus were examined using unbiased stereological techniques and subtle but distinct effects of TCAB exposure were observed. At PND21 (Fig. 11A), TCAB exposure was not found to significantly alter regional volumes of the DG [F(3,38)=2.12; p=0.11] or the CA1 [F(3,40)=0.99; p=0.4]. This absence of an effect was observed in all dose groups. However, an ANOVA of the total number of GCs [F(_3,40_)=2.77; p<0.05] and subsequent post-hoc analysis indicated a significant decrease in cell number in the 10 mg/kg/day TCAB dose group, (Dunnett’s test; p<0.05). Previous work demonstrated a decrease in CA1 pyramidal neurons following developmental TH disruption and T4 deficits [39]. An overall ANOVA of the total number of CA1 PyCs showed a main effect of TCAB exposure [F(_3,40_)=4.626; p<0.007] with a significant decrease observed in the 10 mg/kg/day TCAB dose group as compared to controls (Dunnett’s test; p<0.01). At this early age, the total volume of the individual GCs or the PyCs (neuronal load) occupying each defined region was not significantly altered by TCAB. These findings suggest a specificity of effects on hippocampal regions as a result of developmental TCAB exposure.

With maturation of the hippocampus, the distinct regions have completed all neuronal migration and cell compaction. Consistent with the observations at PND21, at PND150 (Fig. 11B), volumes of the DG and the CA1 regions were not found altered by TCAB (0.1 and 1.0 mg/kg/day) exposure. While no alterations were observed at these dose levels at PND21, when examined at PND150 the total number of GCs was found to be significantly altered by TCAB exposure [F(_2,27_)=3.601; p<0.04]. Significantly fewer cells were observed in the 1.0 mg/kg/day TCAB dose group (Dunnett’s test; p<0.05). No effects of TCAB were observed in CA1 PyC number.

Given that we observed no indication of neuronal death at PND21, one possibility for the age- related loss of GCs following developmental TCAB exposure would be the lack of normal accumulation of dentate granule neurons, similar to what has been previously reported with early postnatal or maternal hypothyroidism [38, 99]. To examine how differences in the 1.0 mg/kg/day dose group could manifest at PND150 but not be present at PND21, changes that occurred with maturation of the hippocampal region were determined [the mean value at PND150 – mean value at PND21 × 100 = %change] (Fig. 11C). Using this formula, the total #GCs showed a minor increase of 3% in controls from PND21 to PND150 while, the change observed in the 1.0 mg/kg/day group represented a 15% decrease with age (p<0.05). The total DG vol increased with age by approximately 10% in the controls, 30% in the 0.1 mg/kg/day group, and 45% in the 1.0mg/kg/day group. The increase in DG vol occurring between PND21 and PND150 was significantly greater in the TCAB dose groups [F(_2,27_)=8.35; p<0.0016] with a significant increase (P<0.001) seen in the 1.0 mg/kg/day dose group. In the CA1, all groups showed a relative change from PND21 to PND150 of approximately 35% decrease in the total #CA1 PyCs. TCAB exposure did not significantly alter the age related decrease in CA1 regional volume with all groups showing between a 5 and 20% decrease with age.

Anatomically, an impact of less dentate granule neurons due to hypothyroidism has been demonstrated by a reduction in the volume of the mossy fiber system and the number of synaptic boutons and synapses within the CA3 synaptic field with developmental PTU exposure [100]. Under these conditions persistent structural alterations in the pre- and postsynaptic compartments of the mossy fibers and reduction of synaptic sites were observed [100]. Other studies examining developmental hypothyroidism and T4 deficits have demonstrated an effect on dentate granule neurons with a deficit in activity dependent processes [101-102] and calcium regulatory proteins [103]. While we did not examine the GC synaptic field in the adult, it is possible that, with decreased DG neuronal number, a deficit in hippocampal mossy fiber synapses occurred in the TCAB-exposed rats.

### 3.6. Cerebellar Morphology and Decreased Purkinje Cell Branching Area

In the developing cerebellum, effects of severe hypothyroidism are characterized by a delayed migration of cerebellar granule cells, a compacted intracellular space associated with reduced axonal growth and dendritic arborization related and a shunting of branching of Purkinje cell dendrites [36, 40, 104-106].

**Figure 12:**
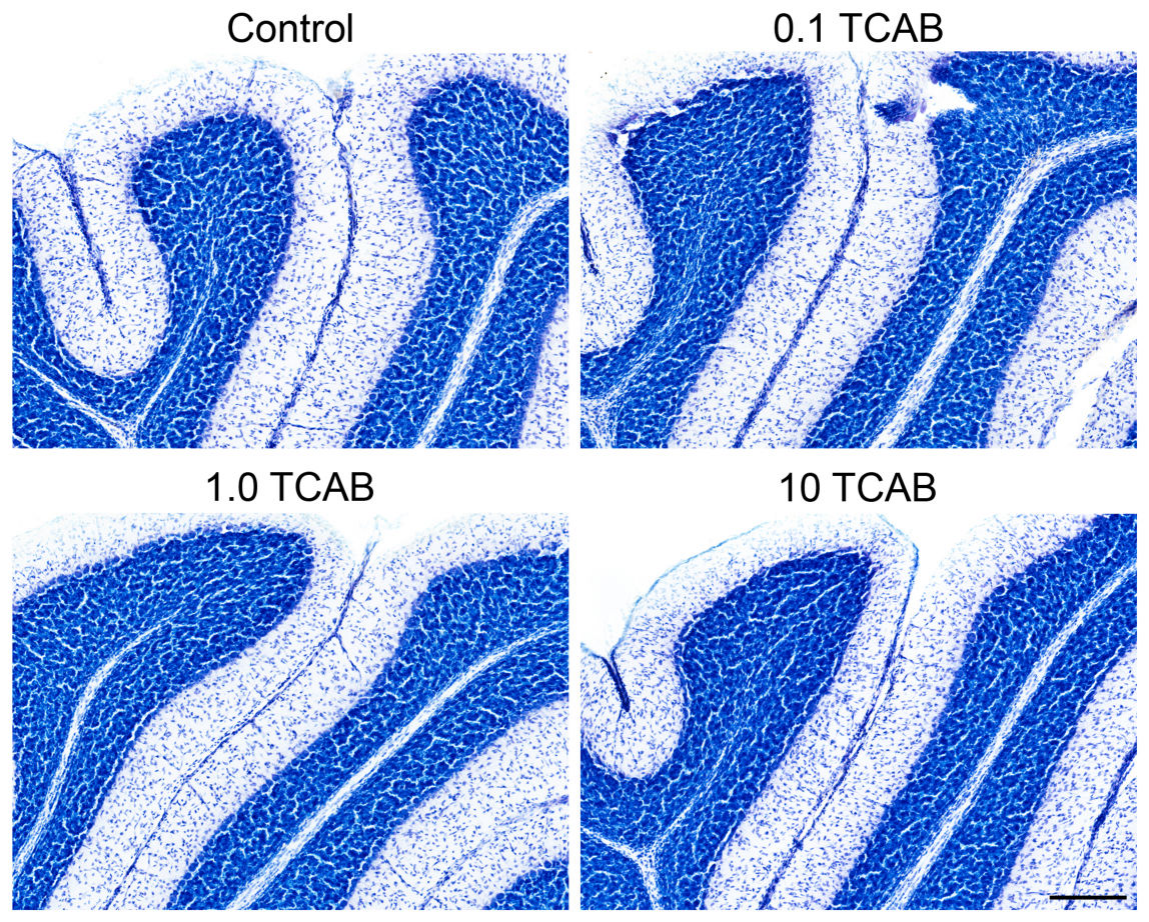
Nissl Staining of Cerebellum at PND21 Representative Nissl staining of the 5^th^-6^th^ cerebellar lobe showing normal distribution of cerebellar granule cells and Purkinje cells at PND21. All animals showed normal development of cerebellar cellular organization and the migration of extracellular granule cells to the intracellular granule cell layer with no evidence of cells remaining in the extracellular granule layer at the primary fissure between folia of lobes. Scale bar [--] = 100 µm.

With maternal hypothyroidism induced by methimazole, at PND25 a small but distinct portion of cells of the external granular cell layer (EGL) are maintained along the primary fissure as compared to the absence of remaining cells in the control [105]. At PND21, we saw no evidence of delayed migration of cerebellar granule neurons, no excess neurons within the migratory zone, and no indication of a residual population of external granule cells along the primary fissure (Fig. 12). However, given the slightly older age of the animals than what is normally examined for such effects we cannot rule out an effect that may have been detected in slightly younger animals.

**Figure 13:**
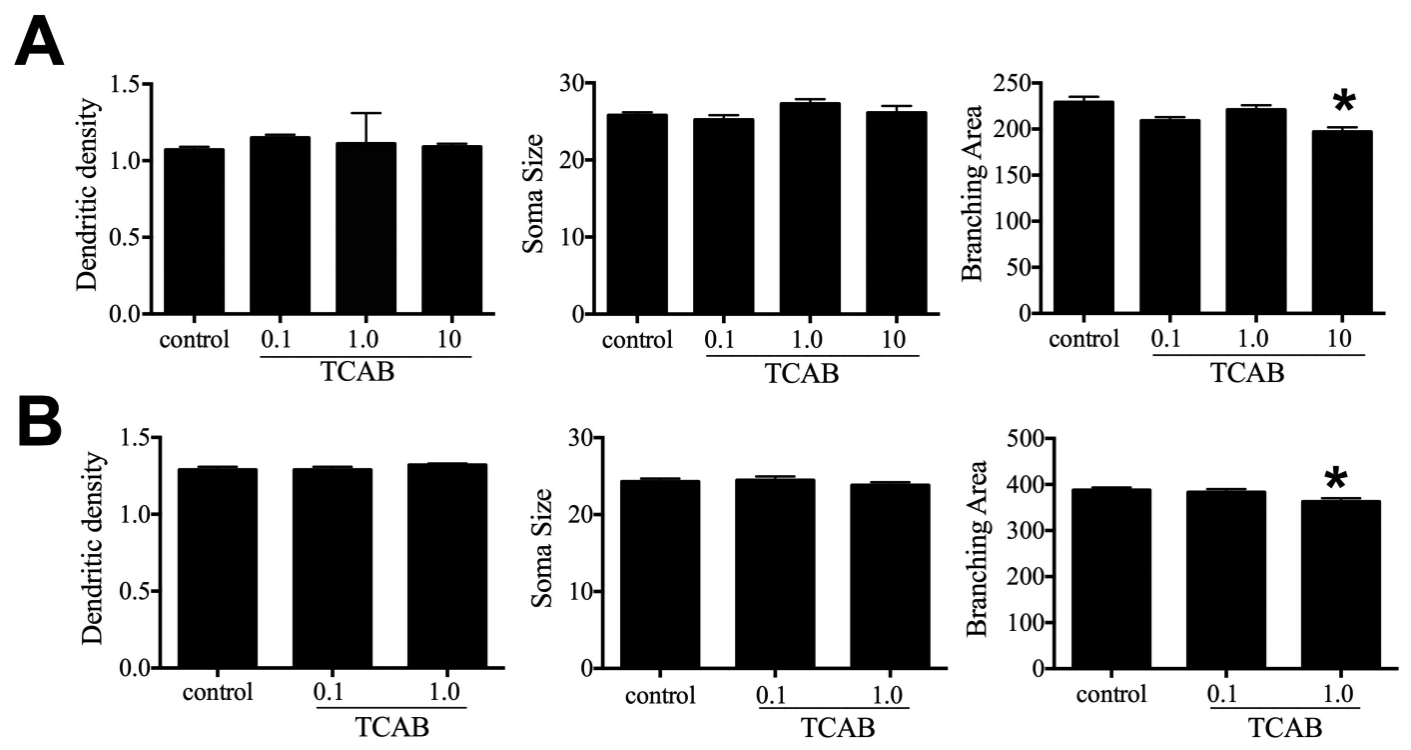
Golgi Staining in Cerebellum Analysis of Golgi staining of cerebellar Purkinje cells at (A) PND21 and (B) PND150 in rats developmentally exposed to TCAB. The mean of 10 neurons within each individual cerebellar section was determined for each animal. Data represent mean +/− SEM. *p<0.05 relative to control.

With regards to cerebellar Purkinje cells, at PND21, Golgi staining showed that dendritic density was not altered by TCAB exposure, with all groups showing a similar level of density as compared to controls (Fig. 13A). In the 1.0mg/kg/day group, Purkinje cell soma size was slightly increased by 10% as compared to controls; however, this failed to reach statistical significance (Fig. 13A). In the 10 mg/kg/day group, the Purkinje cell dendritic branching area was significantly decreased (p<0.05) as compared to controls. In the adult at PND150, no differences were observed between groups (controls, 0.1 and 1.0 mg/kg/day) in Purkinje cell dendritic density or soma size. While an effect on Purkinje cell dendritic branching area was not observed in the 1.0 mg/kg/day group at PND21, a significant decrease (p<0.05) was observed in the adult following maturation of the cerebellum (Fig. 13B).

When the anatomical data was evaluated within a framework of changes occurring with maturation, we found a normal process in control rats of increasing branching area (68%; p<0.0001) and a progression for greater dendritic complexity with a 20% increase in dendritic density (p<0.0001). This would be consistent with previous work demonstrating that Purkinje cell dendrites undergo growth and remodeling post-weaning [107]. In controls, this increase in arborization occurred in the absence of any increase in neuronal soma size. The Purkinje cell branching index (PCBI) is a method to integrate branching area data and dendritic branch density data into a single index. This approach served to differentiate large, sparsely branched Purkinje cells from the same total amount of dendritic branching in a smaller, more heavily branched Purkinje cell. Using this index, we confirmed the expected increase as a function of maturation (100%; p<0.0001). In an examination of the maturational progression of Purkinje cell arborization, we found a similar pattern of changes in the 0.1 mg/kg TCAB dose group when comparisons were made between PND21 and PND150. In this group, the soma size was not found to differ across the two ages. There was an age related increase in branching area of 84% (p<0.0001) that was similar to the increase seen in controls. The dendritic density was significantly increased by 11% (p<0.01). The PCBI was significantly increased with age (105%; p<0.0001) similar to that seen in controls. On visual inspection, Purkinje cells in the PND150 rats that received 1.0 mg/kg TCAB showed a combination of normal appearing cells having well-branched arbors; however, many cells could be found with thickened primary dendritic branches and/or a disorganized branching within the dendritic domain that were not observed in the other two groups that may warrant further investigation (data not shown). In this dose group, soma size was found to be significantly smaller (p<0.0006) in adults as compared to the same dose group at PND21. Even with the smaller soma, significant increases (p<0.0001) were observed in the branching area (68%), dendritic density (18%), and PCBI (98%) in the PND150 rats as compared to PND21 dose-matched animals. Overall, these data suggested that early developmental effects of TCAB exposure and T4 deficiencies continued to impact cell growth and maturation. The impact of less dendritic growth, even marginal, has the potential to alter the development of appropriate axo-dendritic connections between Purkinje cells and cerebellar granule neurons [106-107].

## 4. Conclusions

In the current study, we demonstrated that developmental exposure to TCAB induced a dose response deficit in serum T4 levels in the dam and weanling pup that was not accompanied by a decrease in T3 or increase in TSH. DNT-related neurobehavioral and neuropathological assessments lacked sensitivity to detect changes induced by developmental exposure to TCAB and hypothyroxinemia with serum T4 deficits of approximately 60%. More targeted examination of neuronal and glial endpoints provided a level of sensitivity to detect changes such as morphological staging of microglia and astrocyte morphology within a framework of development, unbiased stereology of the hippocampus, and Golgi staining of Purkinje cells. While Golgi staining and unbiased stereology would require significant changes to the standard DNT neuropathology evaluation, the inclusion of microglia and astrocyte morphological assessments within a framework of developmental staging would be compatible with current methods. Importantly, examination of glia maturation could enhance a DNT evaluation by examining neural cell populations that are known to have a significant influence on brain development. In addition, if the effects observed on glia were related directly to the T4 deficit and not specific for TCAB, inclusion of special stains and morphologically phenotyping may offer an approach to evaluate neurotoxicity of chemical-induced hypothyroxinemia.

## Supplementary Material

8F70125A73BE8B05FCE5B605B86D6593
