# Supplementary material for: Developmental neurotoxicity of 3,3’,4,4’-tetrachloroazobenzene with thyroxine deficit: Sensitivity of glia and dentate granule neurons in the absence of behavioral changes
Source: Toxics. Author manuscript; Available in PMC 2015 May 27. (PMC4445902; doi:10.3390/toxics2030496)
Supplement: 8F70125A73BE8B05FCE5B605B86D6593 [file NIHMS684656-supplement-8F70125A73BE8B05FCE5B605B86D6593.doc]

**SUPPLEMENTAL MATERIAL**

**Developmental neurotoxicity of Tetrachloroazobenzene and thyroxine deficit: Sensitivity of anatomical endpoints in the absence of behavioral changes**

G. Jean Harry, Michelle J. Hooth, Molly Vallant, Mamta Behl, Gregory S. Travlos, James L. Howard, Catherine J. Price, Sandra McBride, Ron Mervis, and Peter R. Mouton

**
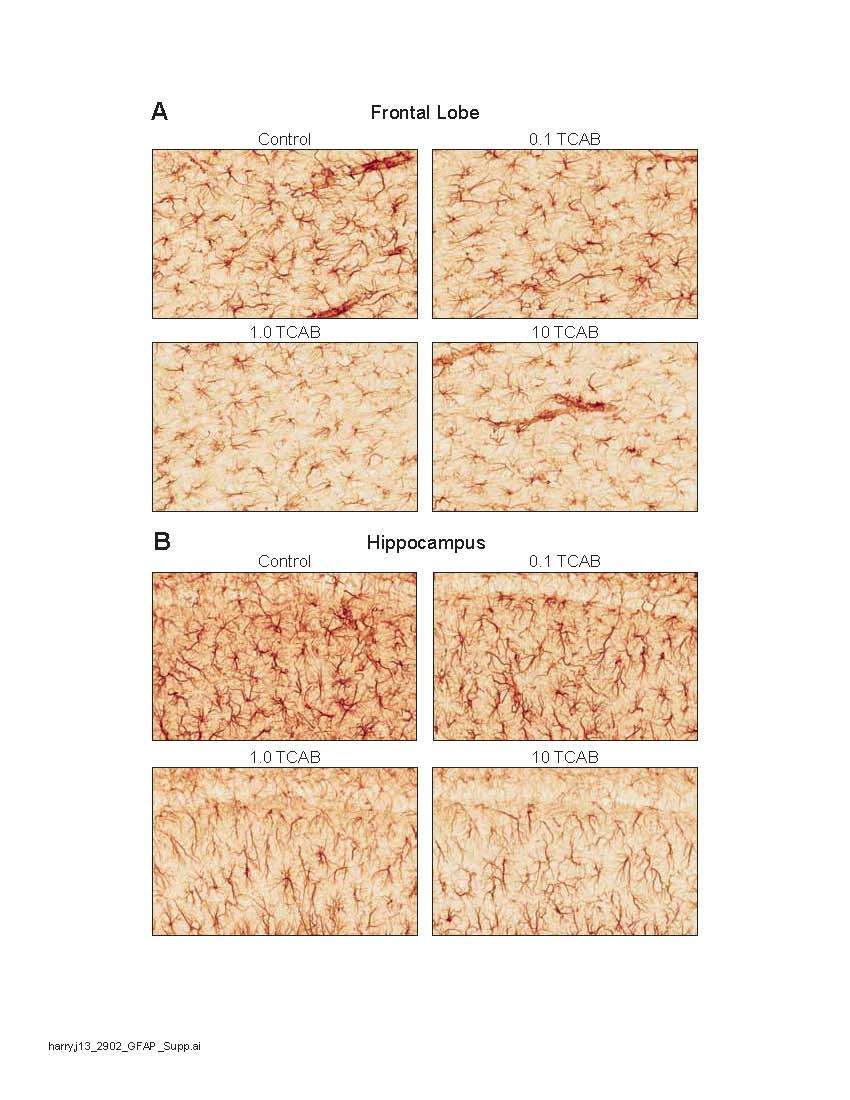
**

**Supplemental Material Figure S1**. Representative GFAP+ astrocytes (brown) at PND21 within the (A) frontal lobe and (B) hippocampal molecular layer (ML). Image provide a larger field of the brain region and identify the region of interest (ROI) for scoring morphology. Scale bar = 50μm.


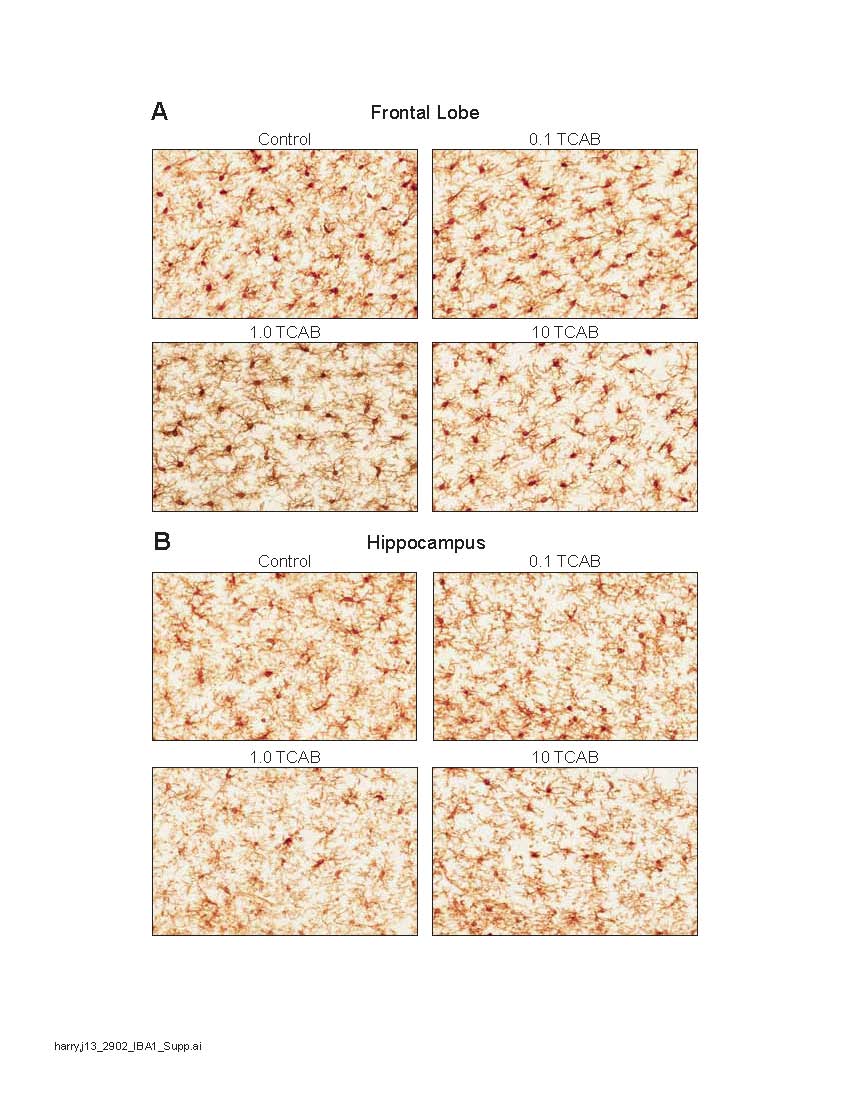


**Supplemental Material Figure S2**. Representative Iba-1+ microglia (brown) at PND21 within the (A) frontal lobe and (B) hippocampal molecular layer (ML). Image provide a larger field of the brain region and identify the region of interest (ROI) for scoring morphology. Scale bar = 50μm.
